# Towards the plastome evolution and phylogeny of *Cycas* L. (Cycadaceae): molecular-morphology discordance and gene tree space analysis

**DOI:** 10.1186/s12870-022-03491-2

**Published:** 2022-03-15

**Authors:** Jian Liu, Anders J. Lindstrom, Xun Gong

**Affiliations:** 1 CAS Key Laboratory for Plant Diversity and Biogeography of East Asia, Kunming Institute of Botany, Chinese Academy of Sciences, 650201 Kunming, Yunnan, China; 2 Department of Economic Plants and Biotechnology, Yunnan Key Laboratory for Wild Plant Resources, Kunming Institute of Botany, Chinese Academy of Sciences, 650201 Kunming, China; 3 Global Biodiversity Conservancy, 144/124 Moo3, Soi Bua Thong, 20250 Bangsalae, Sattahip, Chonburi Thailand; 4 University of Chinese Academy of Sciences, 100049 Beijing, China

**Keywords:** Cycads, *Cycas*, Gene tree discordance, Plastid phylogenomics, Plastome evolution

## Abstract

**Background:**

Plastid genomes (plastomes) present great potential in resolving multiscale phylogenetic relationship but few studies have focused on the influence of genetic characteristics of plastid genes, such as genetic variation and phylogenetic discordance, in resolving the phylogeny within a lineage. Here we examine plastome characteristics of *Cycas* L., the most diverse genus among extant cycads, and investigate the deep phylogenetic relationships within *Cycas* by sampling 47 plastomes representing all major clades from six sections.

**Results:**

All *Cycas* plastomes shared consistent gene content and structure with only one gene loss detected in Philippine species *C. wadei*. Three novel plastome regions (*psb*A-*mat*K, *trn*N-*ndh*F, *chl*L-*trn*N) were identified as containing the highest nucleotide variability. Molecular evolutionary analysis showed most of the plastid protein-coding genes have been under purifying selection except *ndhB*. Phylogenomic analyses that alternatively included concatenated and coalescent methods, both identified four clades but with conflicting topologies at shallow nodes. Specifically, we found three species-rich *Cycas* sections, namely *Stangerioides*, *Indosinenses* and *Cycas*, were not or only weakly supported as monophyly based on plastomic phylogeny. Tree space analyses based on different tree-inference methods both revealed three gene clusters, of which the cluster with moderate genetic properties showed the best congruence with the favored phylogeny.

**Conclusions:**

Our exploration in plastomic data for *Cycas* supports the idea that plastid protein-coding genes may exhibit discordance in phylogenetic signals. The incongruence between molecular phylogeny and morphological classification reported here may largely be attributed to the uniparental attribute of plastid, which cannot offer sufficient information to resolve the phylogeny. Contrasting to a previous consensus that genes with longer sequences and a higher proportion of variances are superior for phylogeny reconstruction, our result implies that the most effective phylogenetic signals could come from loci that own moderate variation, GC content, sequence length, and underwent modest selection.

**Supplementary Information:**

The online version contains supplementary material available at 10.1186/s12870-022-03491-2.

## Background

Due to its lack of recombination, usually uniparental inheritance, and large copy numbers in plant cells [1, 2], the plastid genome has been widely applied in phylogenomics for rebuilding the plant tree of life in the recent decade [3–5]. It has also facilitated the progress of resolving deep relationships of particularly recalcitrant lineages, such as those that have undergone recent radiations [6, 7]. Besides, a complete plastome can also provide insight into the molecular evolutionary patterns including variation in small random repeats, gene rearrangements, duplication, and loss [8–10]. Thus it offers a unique opportunity to better understand plant evolution and is an independent test of hypotheses generated by traditional means [11].

Even though the plastid genome normally duplicates and is inherited as a single locus, recent studies revealed the plastid genes are normally under different selection pressures and produce incongruent trees [12, 13]. The genetic characteristics of plastid genes, such as evolutionary rates, genetic variation, and phylogenetic informativeness, may vary among different genes or functional gene groups and are of great importance in understanding the plastome evolution and phylogenetic inference [13]. However, the effect of genetic characteristics in plastome evolution and resolution of phylogeny remains understudied. Further data exploration like testing phylogenetic signals across genes and using different tree-inferring methods [14, 15] may help to understand the underlying reasons for the conflicting signal of plastid genes but relevant studies remain insufficient. Particularly, questions like the inconsistency of gene tree and species tree topology revealed by the same set of data remain challenging and are involving heat debates [16, 17]. Some recent studies investigating the landscape of plastid gene trees found genes with a longer length and greater genetic diversity to be more powerful in resolving phylogenies [12, 18], while this hypothesis has not been tested across a wide range of groups.

*Cycas* L. is the sole genus of Cycadacace and is the most diverse genus among extant cycads. It compromises approximately 120 species that are distributed in tropical and subtropical Asia, Australia, and Africa, with the highest diversity in Southeast Asia and Australia [19, 20]. The relationship within the genus *Cycas* is contentious regarding the monophyly of several taxonomical sections. Previous phylogenetic studies on *Cycas* supported the classification of this genus into six Sects. [21, 22] according to morphology [23], except for the non-monophyletic *Cycas* section *Stangerioides* [21]. A recent molecular age dating study employed plastomic data to resolve the phylogeny of *Cycas* and found the significance of plastomes in revealing geographical-associated phylogenetic clades while failing to correspond to morphology for some infrageneric Sect. [24]. The previously mentioned study also uncovered there were some inconsistencies between phylogenies based on whole plastomic data and protein-coding genes. However, the reasons accounting for the conflicted topologies between different datasets remain unclear. This framework enables us to test whether genes with contrasting genetic characteristics show incongruent phylogenetic resolution, and what features are in the gene cluster that performs best in revealing the phylogenetic relationship.

In the present study, by using plastomic data from all six sections of *Cycas* representing all major revealed clades as inferred by a phylogeny based on a nearly-complete sampling [21], we reconstructed the plastomic phylogeny and analyzed the chloroplast genome characteristics and the evolutionary rates to (1) investigate the backbone relationships within *Cycas* and evaluate the consistency of gene tree and species tree, (2) uncover the mechanism (e.g., gene tree conflicts, nucleotide substitutions, and evolutionary rates) underlying the challenges in reconstructing a well-resolved phylogeny within Cycadaceae lineages that have experienced historical massive extinctions and (3) gain insights into the growing body of plant plastome evolution, including structural variation, identification of informative markers and selection pressures on functional gene groups.

## Methods

### Plastome sampling and structure characterization

We sampled 47 complete plastomes of *Cycas* from all six sections representing all major clades from a previously published phylogeny [21]. Sampling information, vouchers, and accessions of all *Cycas* materials used in this study can be found in Table S1. Nine genera (Zamiaceae) from Cycadales and two *Ginkgo* (Ginkgoaceae) accessions were used as outgroups based on previous publications [25, 26], yielding a total of 58 taxa incorporated in the present study. We applied both the PGA pipeline [27] and GeSeq [28] to annotate *C. aenigma* (MZ339189) and then used this accession as a reference to perform a uniform plastome annotation for the rest species. The annotations were compared, verified, and adjusted in Geneious prime v.2020 [29]. The visualization of the plastome graph was conducted in OGDRAW [30].

To identify regions with substantial variability across different sections within the genus *Cycas*, we chose a total of 11 accessions representing all six *Cycas* sections. We initially compared the global alignment of the complete chloroplast genomes using mVISTA [31], with *C. aenigma* as a reference. We used the *IRscope* script [32] in R v.3.6.3 [33] to generate and compare the variation of inverted-repeat (IR) and single-copy (SC) borders of the surveyed *Cycas* plastomes. Later we used the *PopGenome* v.2.7.5 package in R to perform the sliding window analysis to assess sequence divergence and determine highly phylogenetically informative sites. The window length was set as 600 bp, with a 100 bp step size. We also calculated and compared the small random repeats (SSR) of sequenced *Cycas* plastomes using the Genome-wide Microsatellite Analyzing Tool Package (GMATA) software [34] with the following search parameters: > 10 repeat units for mononucleotide, > 5 repeat units for dinucleotide, > 4 repeat units for trinucleotide, and > 3 repeat units for tetranucleotide, pentanucleotide, and hexanucleotide SSRs.

### Phylogenetic analyses

We conducted both concatenated and coalescent analyses for phylogenetic inference. Concatenated analyses were implemented in IQTREE v.2.1.1 [35] to infer the maximum likelihood (ML) tree using the ultrafast bootstrap approximation method [36] with 1000 replicates. We inferred the ML trees based on two datasets: concatenated protein-coding genes (PCGs) and the whole plastomic (WP) dataset. The best substitution model determination was simultaneously implemented in ModelFinder [37] under the default Bayesian information criterion (BIC) during the maximum likelihood inference in IQTREE. We also used Bayesian Inference (BI) approach, implemented in MrBayes v.3.2.1 [38], to reconstruct the phylogeny based on the concatenated PCGs and WP datasets for comparisons. The best substitution models (Table S2) of the two concatenated datasets for BI were determined by PartitionFinder v.2.1.1 [39]. For the BI analysis, four simultaneous Markov chain Monte Carlo chains (three cold chains and one hot chain) were implemented, with each run was initiated with one random tree and sampled every 1000 generations for 2*10^7^ generations. The first 25% of trees from each run were discarded as burn-in. All of the tree files generated were visualized in the R package *ggtree* v.3.0.4 [40].

For the coalescent approach, we initially inferred individual gene trees separately for 47 *Cycas* taxa using *Zamia furfuracea* as outgroup in RAxML v.8.2.11 [41], under the “GTRGAMMA” model by resampling a bootstrap of 1000 replicates for each run. The gene trees branches were collapsed when bootstrap support was lower than 33% to reflect uncertainty in gene tree estimates [42]. The collapsed gene trees were then input into ASTRAL-III v.5.6.2 [43] to estimate the species tree with node supports calculated using local posterior probabilities (LPP), which estimates relative quartet support on each branch. Additionally, to assess the level of plastid gene tree conflict for each node of a species tree, we conducted a bipartition analysis using PhyParts (bitbucket.com/blackrim/phyparts) [44] to calculate how many genes are concordant, conflict, or without information for the bipartition in the species tree. Pie charts on the species phylogeny showing the percentage of concordance, percentage in the top alternative bipartition, other conflicting topologies, and uninformative genes were implemented by the python script *phypartspiecharts.py* (Available at https://github.com/mossmatters/phyloscripts/tree/master/phypartspiecharts).

### Nucleotide substitution rate estimation among different groups

To estimate the selective pressure of *Cycas* lineages across its evolutionary history, we concatenated the common PCGs across all 58 taxa to generate a dataset with 56,208 characters after deleting gaps, in which 9,640 sites (17%) are informative. We then estimated the synonymous (dS) and nonsynonymous (dN) substitution rates of the examined taxa using the codeml program of PAML v.4.9 [45] based on this matrix by using *Zamia furfuracea* as reference. The phylogeny generated by IQTREE based on whole plastomic data was used as a constraint tree. The pairwise dN and dS substitution rates between different taxa were calculated based on the custom selection model by setting *CodonFreq* prior as F3 × 4 model. The dN/dS ratio was then calculated for each accession and further compared between pairwise groups. Other parameters remained as default. We used a t-test to detect if there are significant differences in dN/dS substitution rates between different groups (i.e., sections or phylogenetic clades).

We also compared the substitution rates at a functional group level for all PCGs to detect evolutionary rate heterogeneity and to represent different selection regimes acting on PCGs. We first consolidated the PCGs into 11 groups (Table S3) and then estimated the substitution rates of each gene in the codeml program implemented in PAML v.4.9 [45]. The “model = 0” option was used for allowing a single dN/dS value to vary among branches. Other parameters in the codeml control file were the same as above. We used nucleotide diversity (π) and the percentage of variability (PV) to represent the genetic variation of PCGs, where the PV of each PCG was estimated by dividing segregating sites (S, the number of variable positions) by the length of genes. Both π and S were calculated in the program DnaSP v.5 [46] using aligned sequences of the PCGs separately.

### Exploration of plastid gene tree landscape

The plastomic data enables us to test whether the lack of strong support in the phylogenetic tree of *Cycas* was caused by conflicting evolutionary signals among plastid genes. To investigate the variation in phylogenetic signal across the chloroplast genes and compare the performance of different phylogenetic methods, we first inferred gene trees for all 82 PCGs using both BI [38] and ML analyses (see above), then we calculated distances between the trees under different methods using the R package TREESPACE v.1.0.0 [47], respectively. Then we plotted the statistical distribution of trees with Robinson-Foulds algorithms [48] and visualized the ordinations by ggplot2 v.2.2.1 [49]. The *rpl33* gene which was absent in *C. wadei* was removed since the TREESPACE package only accepts gene trees containing the same tips. We also included two ‘species trees’ inferred from concatenated and coalescent analyses based on all PCGs. In total, the dataset consisted of 82 gene trees from 47 taxa and two species trees. We then used the distance between gene trees and the coalescent species tree to estimate gene-tree discordance (GD) of plastid genes. Distances were calculated using the first two PCoAs estimated by TREESPACE. The identification of clusters of trees is based on boundaries between tree topologies [50], and terraces in the phylogenetic tree space [51], which define regions of the tree space inside which trees are closely related through their topology in TREESPACE [47].

After the identification of gene clusters, we then compared the GC content, aligned length, the proportion of variation, and dN/dS of these clusters to explore potential biological interpretations, as these factors may be related to the clustering of gene trees [12, 52]. A simple t-test was again employed to evaluate whether the difference is significant between clusters. Besides, we also conducted a TREESPACE analysis for the 11 functional groups of PCGs (Table S3) to explore their performances in resolving the phylogeny. To identify the best set of genes for the phylogenetic inference of *Cycas* using a more comprehensive sampling in the future, we concatenated genes in each functional group, as well as the three gene clusters recognized by the phylogenetic tree space analysis, to reconstruct phylogenies using rapid bootstrapping RaxML with 1,000 bootstrap replicates.

## Results

### Characteristics of *Cycas* plastomes

All *Cycas* plastomes display the typical quadripartite structure (Fig. S1) with the range of the length from 161,632 bp (*Cycas wadei*) to 163,403 bp (*C. taitungensis*) (Table S1). Most plastomes show a total of 87 PCGs (four located in IR) while the *rpl33* gene is found to be lost in *C. wadei* (Figs. S1 and S2). Plastomes of Cycadaceae are highly conserved with only one trivial event of IR contraction occurring in the sections *Asiorientales* and *Panzhihuaenses* (i.e., *C. revoluta* and *C. panzhihuaensis* in Fig. S3), where IR regions contract from the *ndh*F gene.

**Fig. 1:**
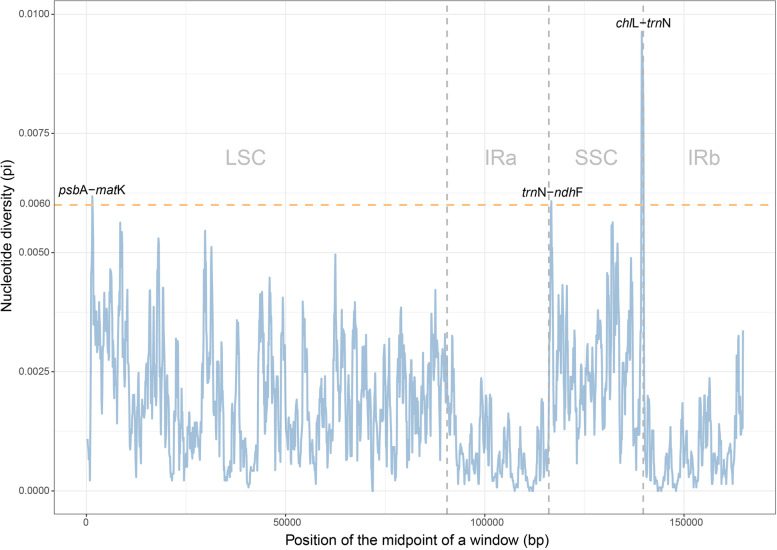
Sliding-window analysis of the whole chloroplast genomes for 47 *Cycas*. The window length is set as 600 bp with the step size as 100 bp. The X-axis denotes the midpoint position of a window. Y-axis shows nucleotide diversity (Pi) of each window. The orange line denotes a Pi threshold of 0.006 to screen high variation regions

Sliding window analysis showed much higher proportions of variable sites in single-copy regions than in the IR regions, especially at the flank boundaries of IRb. Three relatively highly variable regions (*psb*A-*mat*K, *trn*N-*ndh*F, *chl*L-*trn*N) were identified from the plastome sequences (Fig. 1). Section *Wadeae* occupy the highest SSRs (>70, Fig. S4A), and mononucleotides T and A are the most abundant in *Cycas* plastomes. The tetranucleotide (ATAG) is even more commonly repeated than mononucleotides (C and G, Fig. S4B).

**Fig. 2:**
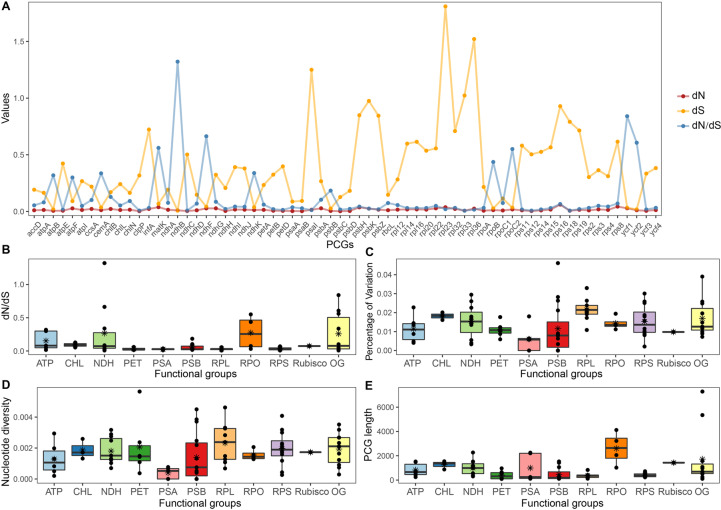
Genetic variation and substitution rates among plastid protein-coding genes (PCGs) and among functional groups. **a** Estimates of nonsynonymous (dN), synonymous (dS) substitution rates and dN/dS of 69 plastid protein-coding genes (PCG) with dN > 0.0003. **b** dN/dS **c** Nucleotide diversity (π) **d** percentage of variation (PV) and **e** gene length of functional groups. Detailed information of functional group is provided in Table S3

Among the 83 genes, *rps16* and *rps8* had relatively higher nonsynonymous (dN) rates, and *rpl23*, *rpl36*, *psa*I, *rpl33*, and *psb*K had higher synonymous (dS) rates (Fig. 2A and Table S4). All PCGs except *ndh*B exhibited considerably low values (< 1) of dN/dS, indicating that most of them have been under purifying selection. The RNA polymerase (RPO) genes and OG had the highest median values of dN/dS (Fig. 2B). Genes that encode subunits involved in photosynthetic processes, such as ATP synthase (ATP), cytochrome b6f complex (PET), and photosystems I and II (PSA and PSB) had lower rates of nucleotide diversity and variations than other functional groups (Fig. 2C and D). In addition, the RPO group had the highest median value of gene length (Fig. 2E).

### Phylogenetic relationships within *Cycas* based on plastomic data

**Fig. 3:**
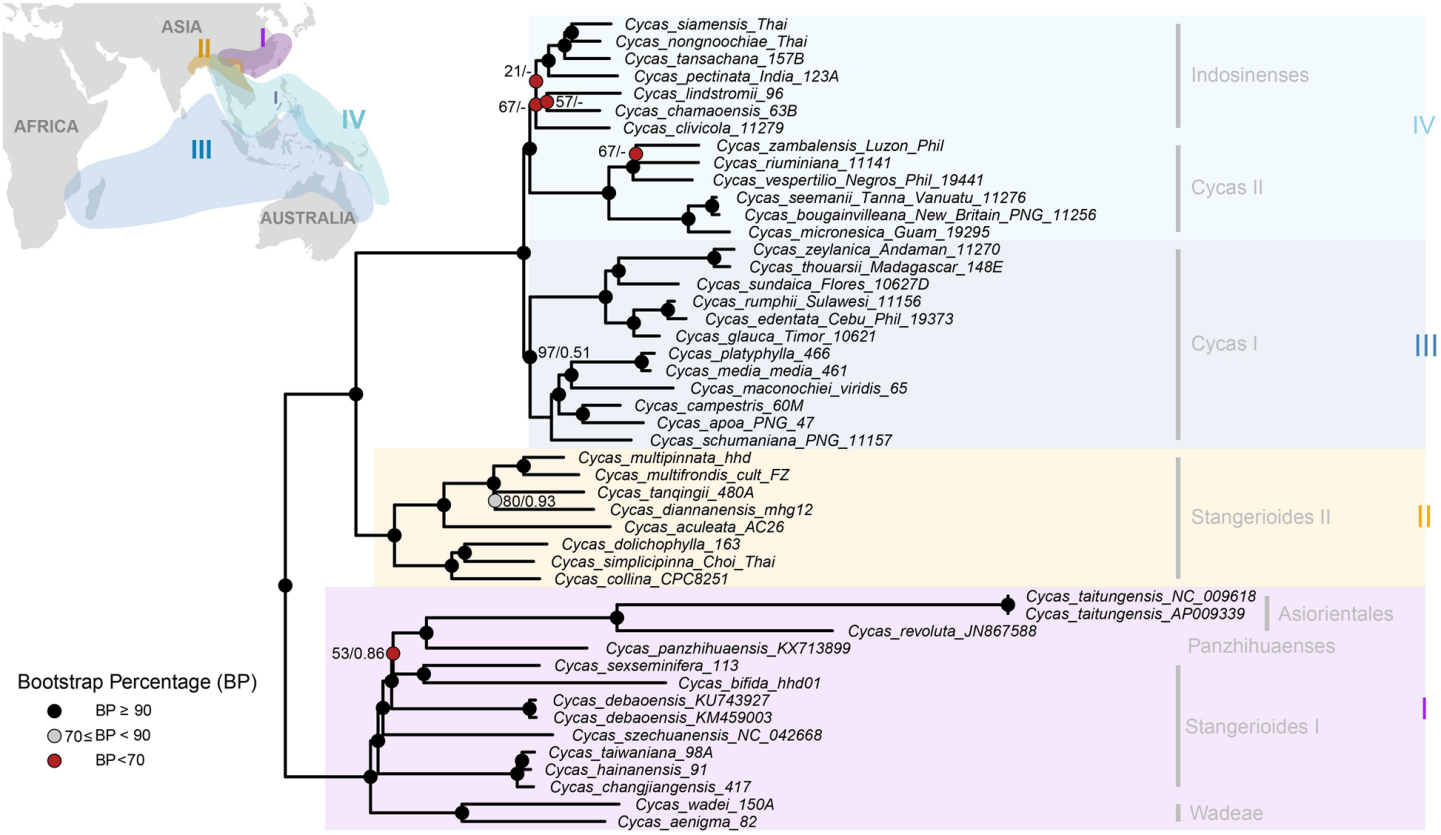
Phylogram of Maximum likelihood (ML) tree of *Cycas* based on full plastomic dataset. Colored dots on the branches represent different bootstrap percentage (BP) ranges indicated at the bottom left. Numbers on the nodes represent BP lower than 90% and corresponded Posterior Probabilities (PP) provided by MrBayes, respectively. The ‘-’ symbol next to BP indicates clade is not supported by Bayesian inference. The inset map depicts the distribution of the revealed clades with corresponded colors

**Fig. 4:**
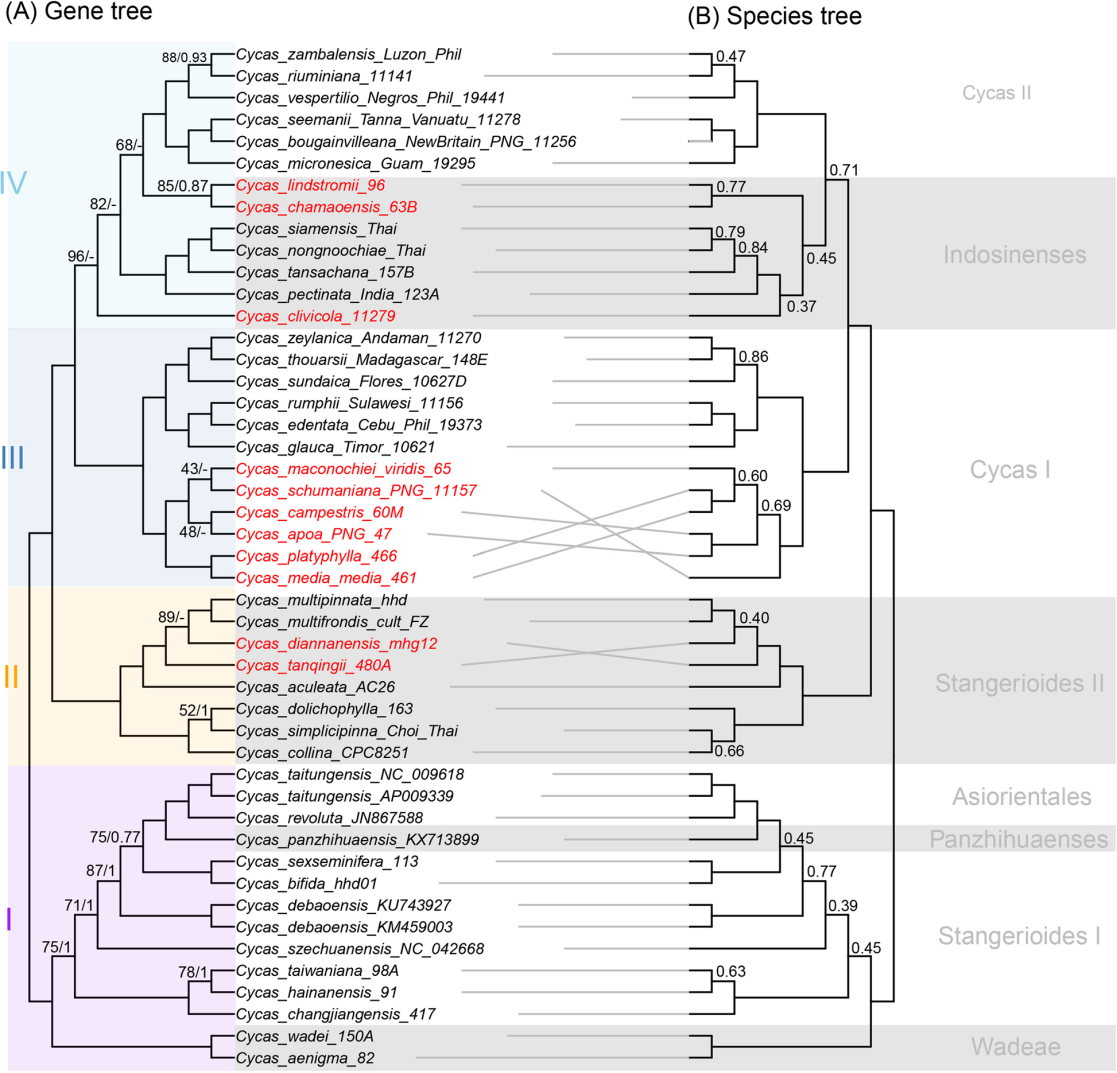
Comparison of tanglegram of *Cycas* based on plastid protein-encoding genes. **a** Maximum likelihood cladogram of *Cycas* based on concatenated genes using IQTREE. Maximum likelihood bootstrap (BS) values and the Posterior Probabilities (PP) calculated from MrBayes are shown at nodes respectively, except nodes with 100% (BS) and 1.0 (PP), ‘-’ indicates no support value. **b** Cladogram generated by the coalescent method in ASTRAL-III. Numbers on the branches depict Local posterior probabilities (LPP), with LPP below 0.9 not shown. Conflicted lineages are highlighted in red font. The highlighted clades I-IV on the left are correspond to Fig. 3 and the subclades corresponded to morphological classification are indicated on the right

Phylogeny inferred by the maximum likelihood method based on the WP dataset generated a well-resolved backbone for *Cycas*, with four major subclades generated and a long branch was found for two accessions of *C. taitungensis* (Fig. 3). There are evident conflicts between the gene tree and the species tree, all shown within the four subclades (Fig. 4). Notably, we found the plastomic data cannot resolve two morphological sections (*Stangerioides* and *Cycas*) as monophyletic (Figs. 3 and 4). Besides, the *Indosinenses* subclade was revealed as unsupported (Fig. 4A) or only weakly supported (Figs. 3 and 4B). The sections *Panzhihuaenses* and *Asiorientales* were resolved as sisters to some species from the section *Stangerioides* but with weak supports in all analyses (Figs. 3 and 4). PhyParts result showed the number of concordant and conflicting genes varied across the species tree, with conflicting signals dominated on some nodes (Fig. S5). However, there is few frequently occurring alternative bipartition against the species tree topology with most are minor or weak supported conflicts.

### Substitution rates estimation and variation of PCGs

**Fig. 5:**
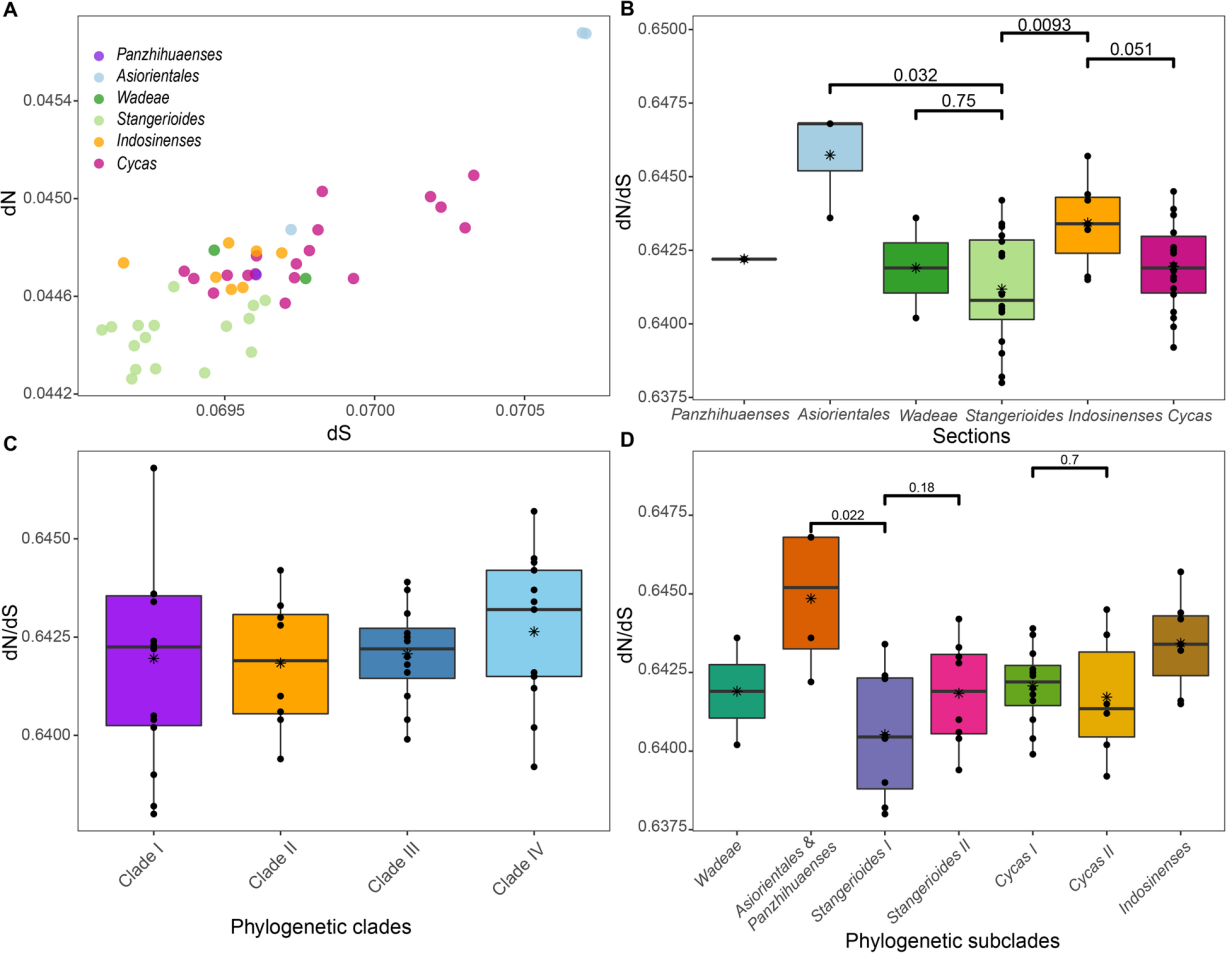
Substitution rates of plastid protein-coding genes in different *Cycas* taxa. **a** The estimations of nonsynonymous (dN), synonymous (dS) substitution rates of 47 *Cycas.* Taxon are grouped by sections; **b** the dN/dS of six sections of *Cycas*, **c** dN/dS of four major phylogenetic clades; **d** dN/dS of seven revealed phylogenetic subclades. See Fig. 3 for the definition of sections and phylogenetic (sub)clades

For the substitution rates of PCGs within *Cycas*, two accessions of *C. taitungensis* from the section *Asiorientales* showed the highest dN and dS. The section *Stangerioides* are relatively slow in nucleotide substitution while some species from the section *Cycas* display higher rates (Fig. 5A). For dN/dS at the section level, *Stangerioides* showed a significantly lower value than the sympatric *Asiorientales*, as well as the section *Indosinenses* (Fig. 5B). Yet there was no significant variation found among four major phylogenetic clades in Cycadaceae (Fig. 5C). We also found no significant dN/dS differences between subclades from the same sections (i.e., *Stangerioides* and *Cycas*), but the clade compromising section *Asiorientales* and *Panzhihuaenses* underwent lower levels of negative (purifying) selection pressure on the plastome compared to its sister clade (*Stangerioides* I, Fig. 5D).

### Phylogenetic tree space analyses

**Fig. 6:**
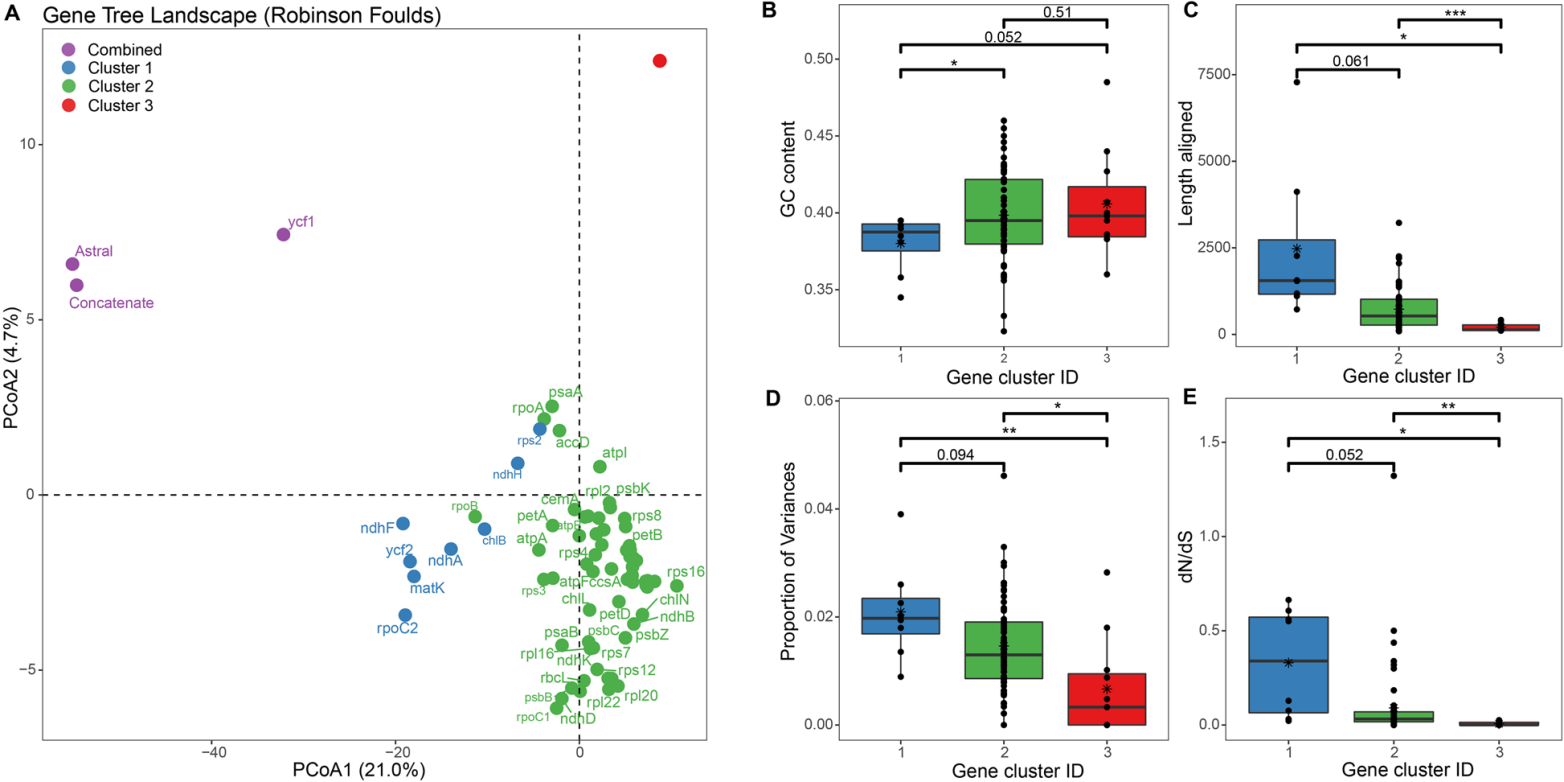
Gene tree clusters revealed by TREESPACE landscape analyses (Robinson-Foulds) based on Maximum Likelihood (ML) tree topologies, and the comparisons of characteristics between the three clusters. **a** Principal coordinate analysis depicting ordinations of species trees versus 82 plastid protein-coding gene trees. Note that there are 11 genes overlapped in Cluster 3 (red dots). The gene names of each cluster can be found in Table 1. **b-e** Boxplots comparing three gene clusters for the variation in phylogenetic signal across the plastid genes, as identified by the TREESPACE analysis. Box-and-whisker plots indicate the median (horizontal line), 25th and 75th percentiles (bottom and top of the box), and limits of the 95% confidence intervals (lower and upper whiskers). Dots beyond the 95% confidence intervals are outliers. The asterisks indicate different levels of significant differences between clusters (*: <0.05, **: <0.01; ***: <0.001). **b** A comparison of the mean GC content of genes among clusters. **c** A comparison of the aligned sequence lengths (numbers of sites) of genes in the three clusters. **d** A comparison of the proportion of genetic variance for genes among clusters. **e** A comparison of the ratio of nonsynonymous to synonymous substitution rates for each gene in the three clusters

**Fig. 7:**
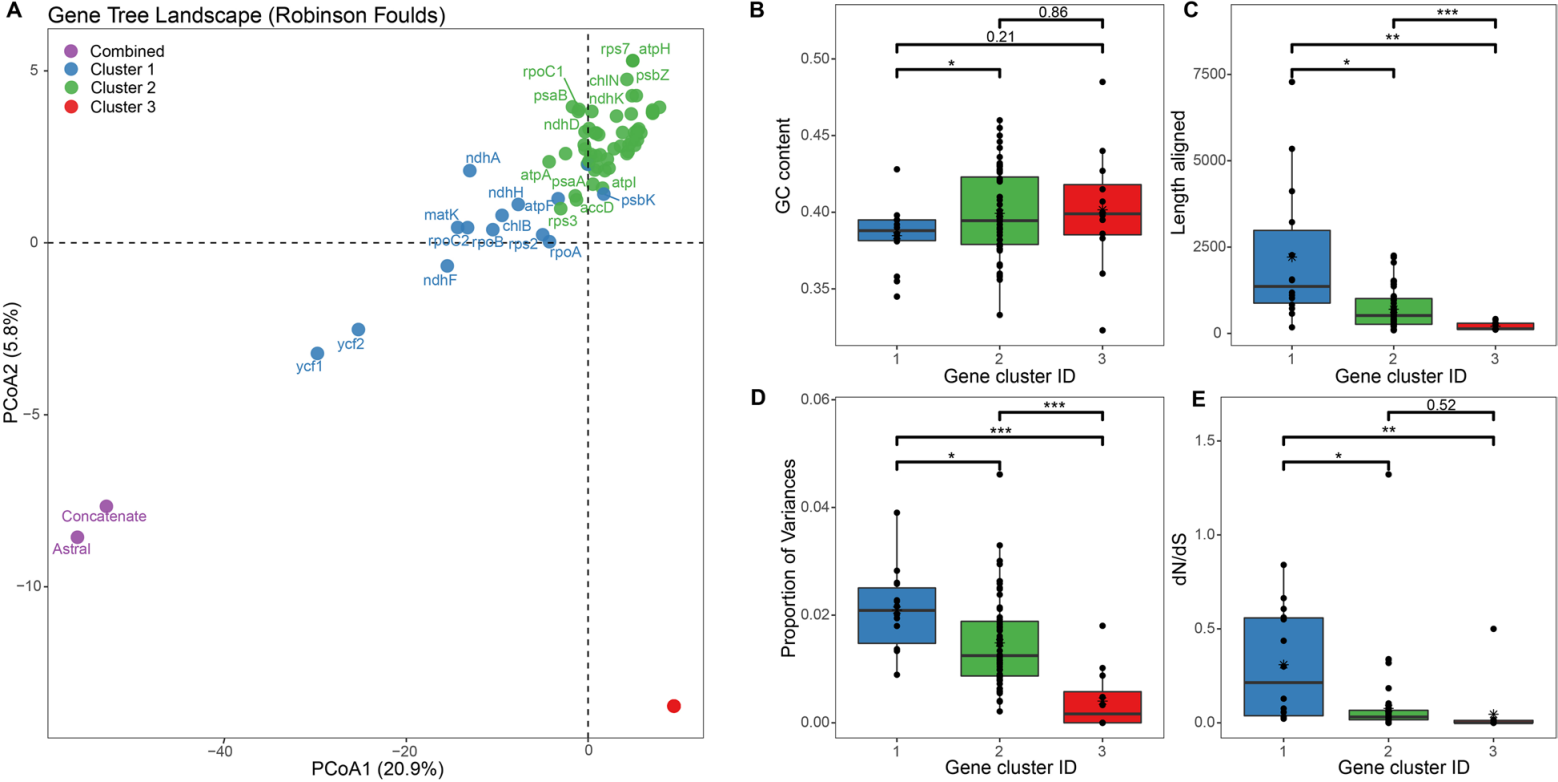
Gene tree clusters revealed by TREESPACE landscape analyses (Robinson-Foulds) based on Bayesian Inference (BI) tree topologies, and the comparisons of characteristics between the three clusters. **a** Principal coordinate analysis depicting ordinations of species trees versus 82 plastid protein-coding gene trees. Note that there are 12 genes overlapped in Cluster 3 (red dots). The gene names of each cluster can be found in Table 1. **b-e** Boxplots comparing three gene clusters for the variation in phylogenetic signal across the plastid genes, as identified by the TREESPACE analysis. Box-and-whisker plots indicate the median (horizontal line), 25th and 75th percentiles (bottom and top of the box), and limits of the 95% confidence intervals (lower and upper whiskers). Dots beyond the 95% confidence intervals are outliers. The asterisks indicate different levels of significant differences between clusters (*: <0.05, **: <0.01; ***: <0.001). **b** A comparison of the mean GC content of genes among clusters. **c** A comparison of the aligned sequence lengths (numbers of sites) of genes in the three clusters. **d** A comparison of the proportion of genetic variance for genes among clusters. **e** A comparison of the ratio of nonsynonymous to synonymous substitution rates for each gene in the three clusters

The tree space analyses based on ML and BI gene trees both revealed three major gene clusters for most PCGs (Fig. 6A and 7A), despite the composition of the genes being different from these two methods to some extent (Table 1). Similarly, both ML- and BI-based tree clustering analyses revealed that gene cluster 2, which showed moderate GC content, aligned length, variation level, and dN/dS in average (B-E in Figs. 6 and 7), displayed the least tree distances between concatenated dataset and gene cluster datasets (Table S5 and Fig. S6). This result further corresponds to the phylogenetic reconstruction based on different concatenated gene clusters, both revealed more similar topologies with concatenated datasets (Figs S7, S3 and S4). An extra TREESPACE analysis also classified the 11 functional gene groups into three clusters (Fig. S8), in which the OG gene tree was closest to the species trees. In contrast, four groups (PET, PSA, PSA, Rubisco) showed weak potential in reconstructing the phylogeny (Fig. S9). However, none of the 11 groups can reflect a well-resolved phylogeny that is congruent to the species tree.

**Table 1:** Gene clusters inferred by different tree inference methods (Maximum Likelihood and Bayesian Inference) and lists of genes in each cluster. Bolded gene names depict distinct genes revealed by two methods in each cluster. Note that the *ycf1* gene is clustered with the combined tree dataset based on the Maximum Likelihood method, thus is not listed here

Gene cluster	Names of genes grouped based on different tree inference methods	
	Maximum Likelihood	Bayesian Inference
Cluster 1 (8 shared)	8 genes: *chlB, matK, ndhA, ndhF, ndhH, rpoC2, rps2, ycf2*	14 genes: ***atpF***, *chlB, matK, ndhA, ndhF, ndhH*, ***psbK***, ***rpl2***, ***rpoA***, ***rpoB***, *rpoC2, rps2*, ***ycf1***, *ycf2*
Cluster 2 (56 shared)	62 genes: *accD, atpA, atpB*, ***atpF***, *atpH, atpI, ccsA, cemA, chlL, chlN, clpP, infA, ndhB, ndhC, ndhD, ndhG, ndhI, ndhJ, ndhK, petA, petB, petD, petL, petN, psaA, psaB, psbA, psbB, psbC, psbD, psbE, psbH, psbI*, ***psbK***, *psbL*, ***psbN***, *psbZ, rbcL*, ***rpl2***, *rpl14, rpl16, rpl20, rpl22, rpl23, rpl32, rpl36*, ***rpoA***, ***rpoB***, *rpoC1, rps12, rps14, rps15, rps16, rps18, rps19, rps3, rps4, rps7, rps8, ycf12, ycf3, ycf4*	56 genes: *accD, atpA, atpB, atpH, atpI, ccsA, cemA, chlL, chlN, clpP, infA, ndhB, ndhC, ndhD, ndhG, ndhI, ndhJ, ndhK, petA, petB, petD, petL, petN, psaA, psaB, psbA, psbB, psbC, psbD, psbE, psbH, psbI, psbL, psbZ, rbcL, rpl14, rpl16, rpl20, rpl22, rpl23, rpl32, rpl36, rpoC1, rps12, rps14, rps15, rps16, rps18, rps19, rps3, rps4, rps7, rps8, ycf12, ycf3, ycf4*
Cluster 3 (11 shared)	11 genes: *atpE, ndhE, petG, psaC, psaI, psaJ, psbF, psbJ, psbM, psbT, rps11*	12 genes: *atpE, ndhE, petG, psaC, psaI, psaJ, psbF, psbJ, psbM*, ***psbN***, *psbT, rps11*

## Discussion

### Gene characteristic diversity in plastomes of *Cycas*

A previous study investigating plastomic variation of three *Cycas* species showed *rpoB*, *psbC*, *ycf1*, *ycf2*, introns of *clp*P, *psb*A*–trn*H, and *trn*L*–trn*F as a great level of interspecific variations and proposed these markers to be useful for DNA barcoding and phylogenetic reconstruction [53]. However, based on a taxa sampling of all major clades from a previous nearly-complete *Cycas* phylogeny [21], our result failed to show that any of the above markers [53] were with the greatest genetic variations. Instead, the three potential regions (*psb*A-*mat*K, *trn*N-*ndh*F, *chl*L-*trn*N) we proposed in the present study should be more practical for future application of the identification of *Cycas* species, highlighting the proposition of potential barcoding markers for intrageneric identification should be based on a broad sampling.

The estimation of nucleotide substitution rates among different genes and different functional groups could provide insight into the diverse selection regimes acting on plastome evolution [13, 54]. Genes encoding subunits involved in photosynthetic processes (e.g., functional groups ATP, PET, PSA, PSB) were previously found to exhibit relatively lower nucleotide diversity and substitution rates than other functional groups of genes in a range group of angiosperms [13, 55, 56]. These patterns (Fig. 2) were also observed in the present gymnosperm lineage Cycadaceae, suggesting the photosynthetic genes are conserved throughout the evolution history of all seed plants. All PCGs seem to display a low level of nonsynonymous substitution rates (dN) across *Cycas*, but we identified a few functional gene groups that have accelerated synonymous substitution rates (dS), mainly the ribosomal protein (RPL and RPS) genes (Fig. 2A). This finding partially follows with the results found in Gentianeae [13], Geraniaceae [54, 57], where the substitution rates for RPL and RPS genes were shown to be highly accelerated in both dN and dS. The low nonsynonymous substitution rate in *Cycas* has also led to a generally low level of dN/dS for all PCGs (Fig. 2A) with only one gene being detected to have undergone positive selection (*ndh*B). Characterized by its relatively longer sequence length, the RNA polymerase (RPO) genes normally occupy a higher level of divergence or substitution rates [13, 58]. However, the RPO genes in *Cycas* plastomes are conserved in sequence variation but showed relatively higher dN/dS than other gene groups. As we found no obvious structure rearrangement detected in *Cycas* plastomes (Figs S2 and S3), the substitution rate variation pattern may be attributed to the heterogeneity of genome-wide mutation rate [13].

### Phylogenetic relationships of *Cycas*

Previous phylogenetic studies of *Cycas* based on nrITS [22] and concatenated nuclear and plastid gene markers [21] both suggested the six sections classified by morphological characters [59] could be justified. However, the phylogeny of *Cycas* based on plastomic data revealed poor or no support for the section *Indosinenses*, and split section *Stangerioides* and section *Cycas* into different subclades or grades corresponding to geography (Fig. 3). As we found no significant selection strength variations among the four major geographical clades in the *Cycas* plastomic phylogeny, the molecular-morphology discordance of *Cycas* may reflect incomplete lineage sorting (ILS) or ongoing introgression/hybridization that have obscured the species phylogeny, as previously documented [21, 60]. While it is difficult to favor one of the above explanations over others only based on plastid data in the present study, further estimates on substitution rates between subclades from sections *Stangerioides* and *Cycas* indicate the divergence of the non-monophyletic sections is not driven by selection (Fig. 5D). The section *Stangerioides* is generally more conservative in substitution rates change and has undergone greater purification selection in their plastomes than its sympatric *Indoensese* and *Asiorientales* taxa. This conservatism in evolutionary rate, along with the long generation of cycads, may account for the slow lineage sorting process in plastomes of section *Stangerioides*. On the contrary, the section *Cycas* showed relatively greater substitution rates and wider geographical distribution (Figs. 3 and 5A), and the non-monophyly of this section may suggest the time for its plastomic lineage sorting is insufficient, or recent hybridization events with section *Indosinenses* during the range expansion for lineages from the section *Cycas*.

The phylogenetic incongruences have long been found across different datasets in *Cycas* [21, 22, 24]. Using a combination of different methods for phylogenetic reconstruction may provide a possible way to evaluate the phylogenetic incongruences [12]. However, here what we found are significant incongruences between gene trees and species trees, which are mostly uncovered at the shallow nodes (where many nodes are not well-supported, Fig. 4). Notably, the species tree synthesized by plastid gene trees, despite suffering from recent complaints [17], generated a more consistent topology with morphological classification than the concatenation method (i.e., the monophyly of Sect. *Indosinense* as resolved in Fig. 4). The prevalent heterogeneity revealed by different methods (BI and ML in concatenation verse coalescence) may suggest: (1) that plastomic data is still insufficient for phylogenetic resolution; and (2) that the PCGs are not tightly linked and behaving as a single locus [14] but are experiencing different evolutionary forces (Fig. 2). Specifically, the single genes used for phylogenetic reconstruction showed conflicting topologies for certain nodes (Fig. S5), resulting in more frequent weakly supported clades only based on PCGs (Fig. 4). It has been noted that using whole plastomic data can offer better support and resolution than PCGs for the phylogeny of *Cycas* [24]. Given the relatively low taxonomic level (within a genus) of this study, it is expected that the relationship resolved by including more molecular data, especially for non-coding regions, would make more sense, as the introduction of more sites into the alignment can potentially reduce stochastic errors [61]. Therefore, we recommend a full plastome dataset in these situations. However, future studies using nuclear phylogenomic data are urgently needed for better understanding patterns of character state evolution and macroevolutionary history for Cycadaceae.

### Potential factors on impacting the phylogenetic resolution

The incongruence of different gene trees may attribute to a host of reasons, such as ILS, introgression, as well as bias in sequencing, alignment, model choice [16, 17]. In the present study, we observed comparable or overwhelming numbers of conflicting genes against the reference tree on some nodes (Fig. S5). Meanwhile, the incongruent phylogenetic signals are extensively detected across the gene trees estimated from both BI and ML methods (Figs. 6 and 7). Previous empirical studies have emphasized the importance of considering variation in phylogenetic signals across plastid genes and the exploration of plastome data (e.g., filter genes for topological concordance) to increase the accuracy of estimating relationships [12, 14, 18]. While using tree space analyses yielded similar results of three gene clusters by both BI and ML methods, the gene clusters based on the BI method identified more genes in Clusters 1 and 3 but less in Cluster 2 than ML method (Table 1). A possible explanation is that despite a threshold of 33% being set for collapsing nodes, the ML trees may still yield more bifurcated topologies (dichotomies) than BI trees, which potentially lead to a generally lower variation range among tree distances between ML trees and species trees (e.g., Table S5). As a consequence, the TREESPACE may show less sensitivity in separating ML trees inferred by genes with higher or lower informative sites.

It is known that genes are not equally useful for reconstructing trees in a given lineage. For *Cycas*, the three gene clusters classified by both BI and ML methods differed greatly in GC content, gene lengths, the proportion of variances, and dN/dS value. Among them, gene clusters 1 and 2 outperformed Cluster 3 which showed the least informative sites and generated the poorest phylogenetic resolution. It also seems that Cluster 1 could be the optimal gene group in reflecting the accurate phylogeny as suggested by three lines of evidence. First, previous studies showed the phylogenetic informativeness of plastid genes were related to sequence length [12, 18], and implied longer genes always perform better and are generally superior for phylogeny reconstruction as revealed by theoretical predictions [62]. Indeed, our study showed gene Cluster 1 tends to have a general greater number of informative characters (statistically insignificant between clusters 1 and 2 for ML method but significant for BI method, Figs. 6 and 7), and the gene trees inferred from these single genes tend to be closer to the trees based on combined data (e.g., *ycf1* and *ycf2* in Fig. 7). Second, a recent study showed loci with dN/dS closest to 1 also tended to support trees in a similar region of tree space as those with high branch supports [52]. This is also consistent with the nature of genes in Cluster 1. Third, previous studies have found that GC-rich regions tend to yield trees with low branch support and have suggested that these regions might be severely affected by long-term recombination rate recombination [63, 64], despite some disputes existing [52]. Analogously, the average GC content level is relatively lower for genes from Clusters 1 than other clusters. All the above characteristics suggested the most suitable genes for phylogenetic reconstruction in *Cycas* could be from Cluster 1.

However, inconsistent with the implications from the above analyses, we found phylogenetic trees based on gene Cluster 2 (Fig. S7), which exhibited moderate sequence lengths as well as other characteristics, were more congruent with the tree reconstructed by the full plastomic data (Fig. 2) and the topologies based on PCGs using both concatenated and coalescence methods (Fig. 3). We further performed maximum likelihood analyses in IQTREE and Bayesian inference to verify the robustness of these topologies. This finding is striking, because the major differences between phylogenetic topologies between gene Clusters 1 and 2 are the placement of sections *Asiorientales* (*C. revoluta* and *C. taitungensis*) and *Panzhihuaenses* (*C. panzhihuaensis*) as well as the position of the section *Wadeae* (*C. wadei* and *C. aenigma*, Fig. S7), three sections that were believed to have undergone vicariance speciation with poor species diversity [24, 59, 65]. The possible explanation for the inconsistent phylogenetic position of these taxa is that the genes from Cluster 1 (at least some of them) have experienced discrepancies or conflict in phylogenetic signals as previously indicated (i.e., due to ILS of independently assorting plastid genes) [14, 52, 66]. Meanwhile, given their faster evolving feature and weaker purification selection strength for gene Cluster 1, the usage of these genes for phylogenetic analyses may have resulted in rapid evolving lineages (i.e., *Asiorientales* + *Panzhihuaenses* clade, see Figs. 3 and 5) to join a distant outgroup (Fig. S7), namely, the notorious long-branch attraction (LBA) effect [61]. Due to this artifact, a more careful selection of sites or gene sequences makes it possible to converge to reliable *Cycas* phylogeny [67]. Consequently, when considering topologies based on three gene clusters, we consider Cluster 2 tree is closest to the best inference of the *Cycas* phylogeny using different datasets and phylogenetic analyses. This is not only because Cluster 2 owned the most genes (56–62 out of 82) representing abundant variation sites, but genes from this cluster may be less affected by the LBA to reflect the true phylogeny.

In addition, we found that all functional gene groups, which merely possessed limited genes from different cluster sources, were not capable to offer sufficient information to infer a robust phylogeny. Particularly, we demonstrated the photosynthesis-involved genes which possessed relatively lower nucleotide diversity and substitution rates (Fig. 2), showed the worst performance. These results, combing together, implied that loci with neither strong nor weak genetic properties have an effective phylogenetic signal, and further highlight the importance of assessing variation in phylogenetic signal across plastid genes. Because in the case of *Cycas*, gene trees frequently conflicted with the species tree, and plastid genes showing longer mean sequence length, weaker negative selection strength, and larger percentage of variations may not necessarily be considered as suitable markers for phylogenetic inference.

### Plastome evolution of *Cycas*

Cycads represent the largest chloroplast genome sizes among all known plastomes of gymnosperms, while are conserved in architecture, gene content, and nucleotide substitution rates [68, 69], reflecting an evolutionary stasis in cycad plastomes [25]. This molecular evolutionary stasis can also be used to explain the conservative nature of *Cycas* plastomes as revealed in our study. Besides the reason from recent radiation, the lineage effect, such as the long generation time, maybe also accountable for the low substitution rates detected in cycads [25]. Thus, despite the comparative plastomic studies for other cycad genera are still lacking, the low-level structural variation across the different *Cycas* sections/species found in this study (Fig. S2), together with the analogous low differentiation level across all cycads [70], implying the universality of infrageneric conservative property for plastomes of other cycad genera.

The only gene loss event across all sampled *Cycas* species was found in *C. wadei*, with the loss of the *rpl33* gene that has been proved to be nonessential for plants under normal conditions but significant in cold stress [71]. Thus, the loss of *rpl33* in *C. wadei* may not affect its viability and growth considering its tropical distribution in the Philippines. Despite the gene loss effect that may have led to its reduced plastome size (the smallest among all samples), it is interesting to note that *C. wadei* is also the species harboring the greatest simple sequence repeats (SSRs). Previous studies suggested that the rise of repeated sequences may play a role in disrupting the functional gene domain (reviewed in [72]) or shaping genome organization [10], but little attention has been paid to the structural effects of SSRs. Combining the Oligocene vicariance event [24] and the plastomic features of *C. wadei*, a possible explanation is that the increasing SSRs may have intervened in the *rpl33* domain, which results in the loss of the unfunctional gene in section *Wadeae* during its evolutionary history, while future studies based on a broader framework to investigate the pattern and mechanisms of this hypothesis are needed.

Microsatellites (SSRs) are useful markers for population genetics, conservation of endangered species, and species delineation for *Cycas* [73–75]. It is not surprising to find that A/T enrichment in SSRs, as this AT preference pattern is widely reported in many plant plastomes [53, 76] and genomes [77]. However, our result suggested that the tetra-nucleotides (e.g., ATAG, TATC, Fig. S4), a nucleotide type often ignored by previous studies, as a major microsatellite component, should be considered as an important source of marker for genetic studies on conservation or reintroduction, species biodiversity assessments in native or introduced areas for Cycadaceae.

## Conclusions

Our study presents a comprehensive plastomic characterization for *Cycas* at species-level, the first-ever reported in extant cycads. Despite a low level of both genetic and structural variation displayed in *Cycas* plastomes, three novel plastid markers (*psb*A-*mat*K, *trn*N-*ndh*F, *chl*L-*trn*N) are identified for DNA barcoding in this genus. Furthermore, we find evidence for rampant phylogenetic discordance of gene trees that may explain the conflict between gene trees and species trees, underlying the difficulties in resolving the phylogeny of *Cycas*, particularly for the shallow nodes. We demonstrate the gene-tree conflict within the plastome can occur at different levels of phylogeny in varying taxa including angiosperm [18], ferns [12], and gymnosperm in this study. We also elucidate those genes with moderate properties (genetic variation, sequence length, dN/dS) may be more suitable for phylogenetic inference in *Cycas*. This highlights the consideration of gene-tree heterogeneity together with comparative investigations of phylogenetic methods in verifying resolving the phylogeny of recalcitrant lineages that have undergone complicated evolutionary histories, such as substitution rate heterogeneity, long massive extinction, and recent diversification. Given the uniparental-inherited nature of plastid genomes, phylogenies relying on plastomic data are still insufficient to disentangle the infrageneric relationships. Future phylogenetic studies on long lifecycle lineages such as cycads should include a much broader sampling of taxa and molecular markers from the nuclear genome, which will contribute to a better understanding of the underlying processes that drive recent radiations in cycad lineages.

## Supplementary Information

**Additional file 1:** **TableS1. **Collectioninformation, morphological classification, vouchers, characteristics andplastome NCBI accessions of the *Cycas* samples used in this study. Allspecimens are identified by the authors of this study (Anders J. Lindstrom,Jian Liu, and Xun Gong). **TableS2. **Thebest nucleotide substitution model for the whole plastome (WP) and the partitioned protein-codinggenes (PCGs) datasetsused in Bayesian Inference as determined by PartitionFinder2. **TableS3. **Plastid genes and functional groups included in the analyses. Genesindicated with asterisk are those with estimated nonsynonymous substitutionrates (dN) lower than 0.0003. **TableS4. **Theestimated substitution rates, nucleotide diversity, aligned length and numberof variable sites (segregates) and percent of variationsof 82 protein-coding genes in 47 *Cycas* of this study. Order is ranked bypercent of variation. **TableS5. **Treedistance between concatenated dataset and gene cluster datasets as inferred bydifferent methods (Bayesian and MaximumLikelihood: ML). Genenames of different clusters can be referred in Table 1. **FigureS1. **Chloroplastgenome graph of *Cycas wadei*. Genes on the outside of the large circleare transcribed clockwise and those on the inside are transcribedcounterclockwise. The genes are color-coded based on their function. The dashedarea represents the GC composition of the chloroplast genome. IR (a & b):inverted repeat region a & b; LSC: large single-copy region; SSC: smallsingle-copy region. **FigureS2. **Globalalignment of 11 *Cycas* genomes and using mVISTA. Alignment was performedusing *C. aenigma* as a reference. Grey arrows above thealignment indicate the orientation of genes. Purple bars represent exons, blueones represent introns, and pink ones represent non-coding sequences (CNS). Acut-off of 50% identity was used for the plots. The Y-scale axis represents thepercent identity within 50–100%. **FigureS3. **Comparison of inverted-repeat (IR) andsingle-copy (SC) borders among 11 *Cycas* chloroplast genomes from sixsections. Gene annotation or portions are represented by colored boxes. JSA:junction between SSC and IRa; JSB: junction between SSC and IRb; JLA: junctionbetween LSC and Ira; JLB: junction between LSC and IRb. **FigureS4. **Thetype and distribution of SSRs in the 47 *Cycas *chloroplast genomes. (a) Theproportion of SSR distribution in different species (b) Number of identifiedSSR motifs in different repeat class types. **FigureS5. **CombinedML topology inferred by ASTRAL (species tree) for *Cycas*, with summary ofconflicting and concordant genes. For each branch, the top number indicates thenumber of homologs concordant with the species tree at that node, and thebottom number indicates the number of homologs in conflict with that clade in thespecies tree. The pie charts at each node present the proportion of homologsthat support that clade (blue), the proportion that support the mainalternative for that clade (pink), the proportion that support the remainingalternatives (orange), and the proportion that inform (conflict or support)this clade that have no bootstrap support (grey). **FigureS6. **Principalcoordinate analyses depicting ordinations of rooted tree topologies (Robinson-Foulds)of four species trees versus six gene-cluster trees. (a): Plots for the firsttwo principal coordinates; (b) Plots for the first and third principalcoordinates. In both plots, a total of 10 trees were obtained from different phylogeneticinference methods (ML: maximum likelihood, BY: Bayesian method) based on allprotein-coding genes (Concatenate_ML: inferred ML tree based onconcatenated genes; Concatenate_BY: inferred Bayesian treebased on concatenated genes; Astral_ML: inferred species tree using ASTRAL-IIIbased on ML gene trees, Astral_BY: inferred species tree using ASTRAL-III basedon Bayesian gene trees), and different gene tree clusters (Clusters 1–3) areplotted. The inset dendrograms reflect the tree distances between the speciestree and the cluster trees revealed by TREESPACE. **FigureS7. **Inferredphylogenies of *Cycas* using RaxML based on different concatenated plastidgene clusters obtained by Maximum likelihood and Bayesian gene trees (clusters1-3, see Table 1 for the genes grouped by different methods). **FigureS8. **Principalcoordinate analyses depicting ordinations of rooted tree topologies (Robinson-Foulds)of two species trees (ASTRAL species tree and concatenated gene tree) versus 11gene trees based on different gene functional groups (see Figure 2 and Table S3 for the group information). (a): Plots for the first two principal coordinates;(b) Plots for the first and third principal coordinates. **FigureS9. **Inferredphylogenies of *Cycas* using RaxML based on different concatenatedfunctional gene groups (see Figure 2 and Table S3 for the information of 11genes groups). The three indicated groups correspond to the clusters revealedin Fig. S8.

## Data Availability

The data of all shown results can be found in the published article and the electronic supplementary material, ordered by their appearance in the figures. The datasets analyzed during the current study are available in the NCBI GenBank repository (See supplementary Table S1 for accessions).
