# Supplementary material for: Towards the plastome evolution and phylogeny of Cycas L. (Cycadaceae): molecular-morphology discordance and gene tree space analysis
Source: BMC Plant Biol. 2022 Mar 15;22:116. doi: 10.1186/s12870-022-03491-2 (PMC8922756; doi:10.1186/s12870-022-03491-2)
Supplement: Supplementary file 1 — Additional file 1: TableS1. Collectioninformation, morphological classification, vouchers, characteristics andplastome NCBI accessions of the Cycas samples used in this study. Allspecimens are identified by the authors of this study (Anders J. Lindstrom,Jian Liu, and Xun Gong). TableS2. Thebest nucleotide substitution model for the whole plastome (WP) and the partitioned protein-codinggenes (PCGs) datasetsused in Bayesian Inference as determined by PartitionFinder2. TableS3. Plastid genes and functional groups included in the analyses. Genesindicated with asterisk are those with estimated nonsynonymous substitutionrates (dN) lower than 0.0003. TableS4. Theestimated substitution rates, nucleotide diversity, aligned length and numberof variable sites (segregates) and percent of variationsof 82 protein-coding genes in 47 Cycas of this study. Order is ranked bypercent of variation. TableS5. Treedistance between concatenated dataset and gene cluster datasets as inferred bydifferent methods (Bayesian and MaximumLikelihood: ML). Genenames of different clusters can be referred in Table 1. FigureS1. Chloroplastgenome graph of Cycas wadei. Genes on the outside of the large circleare transcribed clockwise and those on the inside are transcribedcounterclockwise. The genes are color-coded based on their function. The dashedarea represents the GC composition of the chloroplast genome. IR (a & b):inverted repeat region a & b; LSC: large single-copy region; SSC: smallsingle-copy region. FigureS2. Globalalignment of 11 Cycas genomes and using mVISTA. Alignment was performedusing C. aenigma as a reference. Grey arrows above thealignment indicate the orientation of genes. Purple bars represent exons, blueones represent introns, and pink ones represent non-coding sequences (CNS). Acut-off of 50% identity was used for the plots. The Y-scale axis represents thepercent identity within 50–100%. FigureS3. Comparison of inverted-repeat (IR) andsingle-copy (SC) borders among 11 Cycas chloropl [file 12870_2022_3491_MOESM1_ESM.docx]

**Supplementary Information**

**Table S1** Collection information, morphological classification, vouchers, characteristics and plastome NCBI accessions of the *Cycas* samples used in this study. All specimens are identified by the authors of this study (Anders J. Lindstrom, Jian Liu, and Xun Gong).

| Species | Belonged section | Distribution locality | Voucher | Total length of the plastome (bp) | Number of PCGs | Number of RNAs | GC content | NCBI accession |  |
| --- | --- | --- | --- | --- | --- | --- | --- | --- | --- |
| *Cycas aculeata* | *Stangerioides* | Vietnam | AC26 | 162003 | 87 | 45 | 39.4 | MZ339196 |  |
| *Cycas aenigma* | *Wadeae* | Palawan, Philippines | 82 | 162140 | 87 | 45 | 39.4 | MZ339189 |  |
| *Cycas apoa* | *Cycas* | Paup New Guinea | 47 | 162056 | 87 | 45 | 39.4 | MZ339182 |  |
| *Cycas bifida* | *Stangerioides* | Guangxi, China | hhd01 | 162128 | 87 | 45 | 39.4 | MZ339195 |  |
| *Cycas bougainvilleana* | *Cycas* | New Britain, | 11256 | 162098 | 87 | 45 | 39.4 | MW713704 |  |
| *Cycas campestris* | *Cycas* | Australia | 60M | 162037 | 87 | 45 | 39.4 | MZ339193 |  |
| *Cycas chamaoensis* | *Indosinenses* | Thailand | 63B | 162000 | 87 | 45 | 39.4 | MZ339184 |  |
| *Cycas changjiangensis* | *Stangerioides* | Hainan, China | 417 | 162224 | 87 | 45 | 39.4 | MZ339176 |  |
| *Cycas clivicola* | *Indosinenses* | Malaysia | 11279 | 161999 | 87 | 45 | 39.4 | MZ339183 |  |
| *Cycas collina* | *Stangerioides* | Vietnam | CPC8251 | 161854 | 87 | 45 | 39.5 | MZ339179 |  |
| *Cycas debaoensis* | *Stangerioides* | NA | NA | 162092 | 87 | 45 | 39.4 | KU743927 |  |
| *Cycas debaoensis* | *Stangerioides* | NA | NA | 162094 | 87 | 45 | 39.4 | KM459003 |  |
| *Cycas diannanensis* | *Stangerioides* | Yunnan, China | mhg12 | 162013 | 87 | 45 | 39.5 | MZ339165 |  |
| *Cycas dolichophylla* | *Stangerioides* | Vietnam | 163 | 161838 | 87 | 45 | 39.5 | MZ339170 |  |
| *Cycas edentata* | *Cycas* | Cebu, Philippines | 19373 | 162099 | 87 | 45 | 39.4 | MW713694 | |
| *Cycas glauca* | *Cycas* | Timor, Indonesia | 10621 | 162099 | 87 | 45 | 39.4 | MZ339181 | |
| *Cycas hainanensis* | *Stangerioides* | Hainan, China | 91 | 162215 | 87 | 45 | 39.4 | MZ339171 | |
| *Cycas lindstromii* | *Indosinenses* | Vietnam | 96 | 162061 | 87 | 45 | 39.4 | MZ339185 | |
| *Cycas maconochiei viridis* | *Indosinenses* | Australia | 65 | 162164 | 87 | 45 | 39.4 | MZ339166 | |
| *Cycas media media* | *Cycas* | Australia | 461 | 162106 | 87 | 45 | 39.4 | MZ339192 | |
| *Cycas micronesica* | *Cycas* | Guam | 19295 | 162086 | 87 | 45 | 39.4 | MW713699 | |
| *Cycas multifrondis* | *Stangerioides* | China | FZMF1 | 161993 | 87 | 45 | 39.4 | MZ339191 | |
| *Cycas multipinnata* | *Stangerioides* | Yunnan, China | hhdmp1 | 162060 | 87 | 45 | 39.4 | MZ339174 | |
| *Cycas nongnoochiae* | *Indosinenses* | Thailand | TNN1 | 162096 | 87 | 45 | 39.4 | MZ339169 | |
| *Cycas panzhihuaensis* | *Panzhihuaenses* | NA | NA | 162470 | 87 | 45 | 39.4 | KX713899 | |
| *Cycas pectinata* | *Indosinenses* | India | 123A | 162086 | 87 | 45 | 39.4 | MZ339190 | |
| *Cycas platyphylla* | *Cycas* | Australia | 466 | 162051 | 87 | 45 | 39.4 | MZ339178 | |
| *Cycas revoluta* | *Asiorientales* | NA | NA | 162489 | 87 | 45 | 39.4 | JN867588 | |
| *Cycas riuminiana* | *Cycas* | Philippines | 11141 | 162101 | 87 | 45 | 39.4 | MZ339167 | |
| *Cycas rumphii* | *Cycas* | Sulawesi, Indonesia | 11156 | 162098 | 87 | 45 | 39.4 | MW713712 | |
| *Cycas schumaniana* | *Cycas* | Paup New Guinea | 11157 | 162002 | 87 | 45 | 39.4 | MZ339186 | |
| *Cycas sexseminifera* | *Stangerioides* | Guangxi, China | 113 | 162107 | 87 | 45 | 39.4 | MW713728 | |
| *Cycas seemanii* | *Cycas* | Tanna, Vanuatu | 11278 | 162093 | 87 | 45 | 39.4 | MZ339172 | |
| *Cycas siamensis* | *Indosinenses* | Thailand | SA1 | 162117 | 87 | 45 | 39.4 | MZ339177 | |
| *Cycas simplicipinna* | *Stangerioides* | Choi, Thailand | NNSI1 | 161961 | 87 | 45 | 39.4 | MZ339180 | |
| *Cycas sundaica* | *Cycas* | Flores | 10627D | 162099 | 87 | 45 | 39.4 | MW713727 | |
| *Cycas szechuanensis* | *Stangerioides* | NA | NA | 162083 | 87 | 45 | 39.4 | NC042668 | |
| *Cycas taitungensis* | *Asiorientales* | NA | NA | 163403 | 87 | 45 | 39.4 | AP009339 | |
| *Cycas taitungensis* | *Asiorientales* | NA | NA | 163403 | 87 | 45 | 39.4 | NC009618 | |
| *Cycas taiwaniana* | *Stangerioides* | Hainan, China | 98A | 162204 | 87 | 45 | 39.4 | MZ339194 | |
| *Cycas tanqingii* | *Stangerioides* | Yunnan, China | 480A | 162060 | 87 | 45 | 39.5 | MZ339173 | |
| *Cycas tansachana* | *Indosinenses* | Thailand | 157B | 162058 | 87 | 45 | 39.4 | MZ339187 | |
| *Cycas thouarsii* | *Cycas* | Madagascar | 148E | 161753 | 87 | 45 | 39.4 | MW713696 | |
| *Cycas vespertilio* | *Cycas* | Negros, Philippines | 19441 | 162105 | 87 | 45 | 39.4 | MZ339188 | |
| *Cycas wadei* | *Wadeae* | Culion, Philippines | 150A | 161632 | 86 | 45 | 39.4 | MZ339175 | |
| *Cycas zambalensis* | *Cycas* | Luzon | NNZM1 | 162085 | 87 | 45 | 39.4 | MZ339168 | |
| *Cycas zeylanica* | *Cycas* | Andaman | 11270 | 161756 | 87 | 45 | 39.4 | MW713697 | |

**Table S2** The best nucleotide substitution model for the whole plastome (WP) and the partitioned protein-coding genes (PCGs) datasets used in Bayesian Inference as determined by PartitionFinder2.

| Datasets | Substitution model |
| --- | --- |
| WP | GTR+G+I |
| PCGs | charset Subset1 = 1-84675\3: GTR+I+G  charset Subset2 = 2-84675\3: GTR+I+G+X  charset Subset3 = 3-84675\3: GTR+I+G+X |

**Table S3** Plastid genes and functional groups included in the analyses. Genes indicated with asterisk are those with estimated nonsynonymous substitution rates (dN) lower than 0.0003.

| Functional groups | Genes |
| --- | --- |
| Photosystem I (PSA) | *psaA, psaB, psaC*, psaI, psaJ** |
| Photosystem II (PSB) | *psbA, psbB, psbC, psbD, psbE*, psbF*, psbH, psbI*, psbJ*, psbK, psbL*, psbM*, psbN*, psbT*, psbZ* |
| Cytochrome B6f complex (PET) | *petA, petB, petD, petG*, petL*, petN** |
| ATP synthase (ATP) | *atpA, atpB, atpE, atpF, atpH*, atpI* |
| Rubisco large subunit (Rubisco) | *rbcL* |
| RNA polymerase (RPO) | *rpoA, rpoB, rpoC1, rpoC2* |
| Ribosomal proteins large subunit (RPL) | *rpl2, rpl14, rpl16, rpl20, rpl22, rpl23, rpl32, rpl33, rpl36* |
| Ribosomal proteins small subunit (RPS) | *rps2, rps3, rps4, rps7*, rps8, rps11, rps12, rps14, rps15, rps16, rps18, rps19* |
| NADH dehydrogenase (NDH) | *ndhA, ndhB, ndhC, ndhD, ndhE*, ndhF, ndhG ndhH ndhI ndhJ ndhK* |
| Chlorophyll synthesis (CHL) | *chlB, chlL, chlN* |
| **Other genes (OG)** |  |
| Conserved coding frame | *ycf1, ycf2, ycf3, ycf4, ycf12** |
| Acetyl-CoA-carboxylase | *accD* |
| ATP-dependent protease | *clpP* |
| Cytochrome c biogenesis | *ccsA* |
| Membrane protein | *cemA* |
| Maturase | *matK* |
| Translational initiation factor | *infA* |

**Table S4** The estimated substitution rates, nucleotide diversity, aligned length and number of variable sites (segregates) and percent of variations of 82 protein-coding genes in 47 *Cycas* of this study. Order is ranked by percent of variation.

| Gene  names | Group assigned | dN  (nonsynonymous) | dS  (synonymous) | dN/dS | Pi | Length (bp) | Segregates | Percent of Variation  (Segregates/Length) |
| --- | --- | --- | --- | --- | --- | --- | --- | --- |
| *psbA* | PSB | 0.0276 | 0.2664 | 0.103604 | 0.00385 | 1062 | 49 | 0.046139 |
| *matK* | OG | 0.0378 | 0.0674 | 0.560831 | 0.00029 | 1563 | 61 | 0.039028 |
| *rpl23* | RPL | 0.037 | 1.8094 | 0.020449 | 0.00326 | 273 | 9 | 0.032967 |
| *rps8* | RPS | 0.043 | 0.6155 | 0.069862 | 0.00229 | 399 | 12 | 0.030075 |
| *ndhG* | NDH | 0.0276 | 0.3235 | 0.085317 | 0.00289 | 543 | 16 | 0.029466 |
| *psbK* | PSB | 0.025 | 0.9754 | 0.025631 | 0.00371 | 177 | 5 | 0.028249 |
| *rpl36* | RPL | 0.0248 | 1.5214 | 0.016301 | 0.00148 | 114 | 3 | 0.026316 |
| *psbH* | PSB | 0.0364 | 0.8487 | 0.042889 | 0.0045 | 228 | 6 | 0.026316 |
| *ndhF* | NDH | 0.0272 | 0.041 | 0.663415 | 0.00317 | 2268 | 59 | 0.026014 |
| *rps3* | RPS | 0.0182 | 0.364 | 0.05 | 0.003 | 657 | 17 | 0.025875 |
| *ycf1* | OG | 0.0279 | 0.0332 | 0.840361 | 0.00318 | 5346 | 138 | 0.025814 |
| *rps19* | RPS | 0.0147 | 0.7145 | 0.020574 | 0.00318 | 279 | 7 | 0.02509 |
| *ccsA* | OG | 0.0221 | 0.2188 | 0.101005 | 0.00164 | 966 | 24 | 0.024845 |
| *rpl32* | RPL | 0.0223 | 0.7093 | 0.031439 | 0.00462 | 210 | 5 | 0.02381 |
| *atpF* | ATP | 0.0272 | 0.0908 | 0.299559 | 0.00294 | 571 | 13 | 0.022767 |
| *ndhA* | NDH | 0.0146 | 0.1918 | 0.076121 | 0.00275 | 1107 | 25 | 0.022584 |
| *rpl2* | RPL | 0.0159 | 0.2823 | 0.056323 | 0.00238 | 831 | 18 | 0.021661 |
| *rpl22* | RPL | 0.0274 | 0.5564 | 0.049245 | 0.00283 | 420 | 9 | 0.021429 |
| *psbZ* | PSB | 0.0158 | 0.8448 | 0.018703 | 0.00132 | 189 | 4 | 0.021164 |
| *chlB* | CHL | 0.0218 | 0.1699 | 0.128311 | 0.00259 | 1542 | 31 | 0.020104 |
| *rpl33* | RPL | 0.0068 | 1.0231 | 0.006646 | 0.00127 | 201 | 4 | 0.0199 |
| *ycf12* | OG | 0.0001 | 1.4361 | 6.96E-05 | 0.00123 | 102 | 2 | 0.019608 |
| *rpoC2* | RPO | 0.018 | 0.0327 | 0.550459 | 0.00206 | 4119 | 80 | 0.019422 |
| *rpl16* | RPL | 0.0183 | 0.6143 | 0.02979 | 0.00331 | 417 | 8 | 0.019185 |
| *rps15* | RPS | 0.0166 | 0.5654 | 0.02936 | 0.00172 | 267 | 5 | 0.018727 |
| *chlL* | CHL | 0.0127 | 0.2416 | 0.052566 | 0.00171 | 873 | 16 | 0.018328 |
| *psaI* | PSA | 0.0148 | 1.2503 | 0.011837 | 0.00077 | 111 | 2 | 0.018018 |
| *rps2* | RPS | 0.0095 | 0.3033 | 0.031322 | 0.00176 | 723 | 13 | 0.017981 |
| *ndhI* | NDH | 0.0168 | 0.3909 | 0.042978 | 0.00248 | 558 | 10 | 0.017921 |
| *petA* | PET | 0.0141 | 0.2335 | 0.060385 | 0.00149 | 963 | 17 | 0.017653 |
| *rpl20* | RPL | 0.0163 | 0.5362 | 0.030399 | 0.00108 | 348 | 6 | 0.017241 |
| *ndhK* | NDH | 0.0126 | 0.0372 | 0.33871 | 0.00123 | 993 | 17 | 0.01712 |
| *chlN* | CHL | 0.0151 | 0.164 | 0.092073 | 0.00132 | 1365 | 22 | 0.016117 |
| *rps16* | RPS | 0.0608 | 0.9291 | 0.06544 | 0.00408 | 255 | 4 | 0.015686 |
| *ndhJ* | NDH | 0.0156 | 0.3795 | 0.041107 | 0.00103 | 522 | 8 | 0.015326 |
| *accD* | OG | 0.0104 | 0.1919 | 0.054195 | 0.00211 | 1080 | 16 | 0.014815 |
| *ndhD* | NDH | 0.0102 | 0.1474 | 0.069199 | 0.00151 | 1503 | 22 | 0.014637 |
| *atpA* | ATP | 0.0132 | 0.1647 | 0.080146 | 0.0013 | 1524 | 22 | 0.014436 |
| *rpoA* | RPO | 0.0068 | 0.2159 | 0.031496 | 0.00133 | 1023 | 14 | 0.013685 |
| *ndhH* | NDH | 0.0045 | 0.2069 | 0.02175 | 0.00131 | 1182 | 16 | 0.013536 |
| *atpI* | ATP | 0.013 | 0.2675 | 0.048598 | 0.00198 | 747 | 10 | 0.013387 |
| *rpoB* | RPO | 0.0117 | 0.0268 | 0.436567 | 0.00128 | 3222 | 43 | 0.013346 |
| *ycf4* | OG | 0.0121 | 0.3829 | 0.031601 | 0.00233 | 555 | 7 | 0.012613 |
| *petB* | PET | 0.0065 | 0.3248 | 0.020012 | 0.00143 | 648 | 8 | 0.012346 |
| *infA* | OG | 0.0223 | 0.7229 | 0.030848 | 0.00267 | 249 | 3 | 0.012048 |
| *ycf3* | OG | 0.0058 | 0.3341 | 0.01736 | 0.00268 | 513 | 6 | 0.011696 |
| *rps4* | RPS | 0.0135 | 0.3112 | 0.04338 | 0.00203 | 606 | 7 | 0.011551 |
| *rpoC1* | RPO | 0.0085 | 0.1143 | 0.074366 | 0.00154 | 2055 | 23 | 0.011192 |
| *petN* | PET | 0.0002 | 1.9105 | 0.000105 | 0.00565 | 90 | 1 | 0.011111 |
| *rpl14* | RPL | 0.0178 | 0.5984 | 0.029746 | 0.00068 | 369 | 4 | 0.01084 |
| *petL* | PET | 0.0001 | 1.3377 | 7.48E-05 | 0.00237 | 96 | 1 | 0.010417 |
| *rps11* | RPS | 0.0099 | 0.5808 | 0.017045 | 0.00043 | 393 | 4 | 0.010178 |
| *rps14* | RPS | 0.0093 | 0.526 | 0.017681 | 0.00221 | 303 | 3 | 0.009901 |
| *clpP* | OG | 0.0024 | 0.3176 | 0.007557 | 0.00074 | 606 | 6 | 0.009901 |
| *rbcL* | Rubisco | 0.0109 | 0.1467 | 0.074301 | 0.00173 | 1428 | 14 | 0.009804 |
| *psbI* | PSB | 0.0001 | 1.489 | 6.72E-05 | 0.00075 | 111 | 1 | 0.009009 |
| *ycf2* | OG | 0.0108 | 0.0179 | 0.603352 | 0.00091 | 7284 | 65 | 0.008924 |
| *petG* | PET | 0.0002 | 1.7923 | 0.000112 | 0.00037 | 114 | 1 | 0.008772 |
| *rps18* | RPS | 0.0062 | 0.7911 | 0.007837 | 0.00118 | 228 | 2 | 0.008772 |
| *atpB* | ATP | 0.0058 | 0.0182 | 0.318681 | 0.00082 | 1485 | 13 | 0.008754 |
| *psbL* | PSB | 0.0001 | 1.0571 | 9.46E-05 | 0.00247 | 117 | 1 | 0.008547 |
| *psbD* | PSB | 0.0041 | 0.182 | 0.022527 | 0.0004 | 1062 | 9 | 0.008475 |
| *rps12* | RPS | 0.0038 | 0.5039 | 0.007541 | 0.0016 | 369 | 3 | 0.00813 |
| *psbE* | PSB | 0.0001 | 0.6341 | 0.000158 | 0.00219 | 252 | 2 | 0.007937 |
| *psbB* | PSB | 0.0037 | 0.0254 | 0.145669 | 0.00073 | 1527 | 12 | 0.007859 |
| *cemA* | OG | 0.0117 | 0.0348 | 0.336207 | 0.00352 | 690 | 5 | 0.007246 |
| *psaB* | PSA | 0.0026 | 0.0929 | 0.027987 | 0.00067 | 2205 | 14 | 0.006349 |
| *psbC* | PSB | 0.0021 | 0.1271 | 0.016522 | 0.00052 | 1422 | 9 | 0.006329 |
| *petD* | PET | 0.0054 | 0.3977 | 0.013578 | 0.00109 | 507 | 3 | 0.005917 |
| *psaA* | PSA | 0.0032 | 0.0872 | 0.036697 | 0.00052 | 2253 | 13 | 0.00577 |
| *ndhC* | NDH | 0.0039 | 0.5022 | 0.007766 | 0.00129 | 363 | 2 | 0.00551 |
| *atpE* | ATP | 0.0067 | 0.4229 | 0.015843 | 0.0002 | 417 | 2 | 0.004796 |
| *atpH* | ATP | 0.0001 | 0.799 | 0.000125 | 0.0005 | 246 | 1 | 0.004065 |
| *ndhB* | NDH | 0.0095 | 0.1132 | 0.083922 | 0.00068 | 1524 | 6 | 0.003937 |
| *psbM* | PSB | 0.0001 | 0.0002 | 0.5 | 0.00044 | 294 | 1 | 0.003401 |
| *ndhE* | NDH | 0.0001 | 0.5419 | 0.000185 | 0.00151 | 303 | 1 | 0.0033 |
| *rps7* | RPS | 0 | 0.4512 | 0 | 0.00026 | 471 | 1 | 0.002123 |
| *psbF* | PSB | 0.0002 | 1.623 | 0.000123 | 0 | 120 | 0 | 0 |
| *psbJ* | PSB | 0.0001 | 1.4169 | 7.06E-05 | 0 | 123 | 0 | 0 |
| *psbT* | PSB | 0.0001 | 1.2913 | 7.74E-05 | 0 | 108 | 0 | 0 |
| *psbN* | PSB | 0.0001 | 1.1346 | 8.81E-05 | 0 | 132 | 0 | 0 |
| *psaJ* | PSA | 0.0001 | 1.0723 | 9.33E-05 | 0 | 135 | 0 | 0 |
| *psaC* | PSA | 0.0001 | 0.9979 | 0.0001 | 0 | 246 | 0 | 0 |

**Table S5** Tree distance between concatenated dataset and gene cluster datasets as inferred by different methods (Bayesian and Maximum Likelihood: ML). Gene names of different clusters can be referred in Table 1.

|  | Concatenate_Bayesian | Concatenate_ML |
| --- | --- | --- |
| Concatenate_Bayesian | 0.00 | 27.26 |
| Concatenate_ML | 27.26 | 0.00 |
| Cluster1_Bayesian | 120.72 | 108.31 |
| Cluster2_Bayesian | 18.71 | 31.51 |
| Cluster3_Bayesian | 343.87 | 329.12 |
| Cluster1_ML | 95.97 | 84.22 |
| Cluster2_ML | 24.22 | 17.61 |
| Cluster3_ML | 85.37 | 95.70 |

**Figure S1** Chloroplast genome graph of *Cycas wadei*. Genes on the outside of the large circle are transcribed clockwise and those on the inside are transcribed counterclockwise. The genes are color-coded based on their function. The dashed area represents the GC composition of the chloroplast genome. IR (a & b): inverted repeat region a & b; LSC: large single-copy region; SSC: small single-copy region.


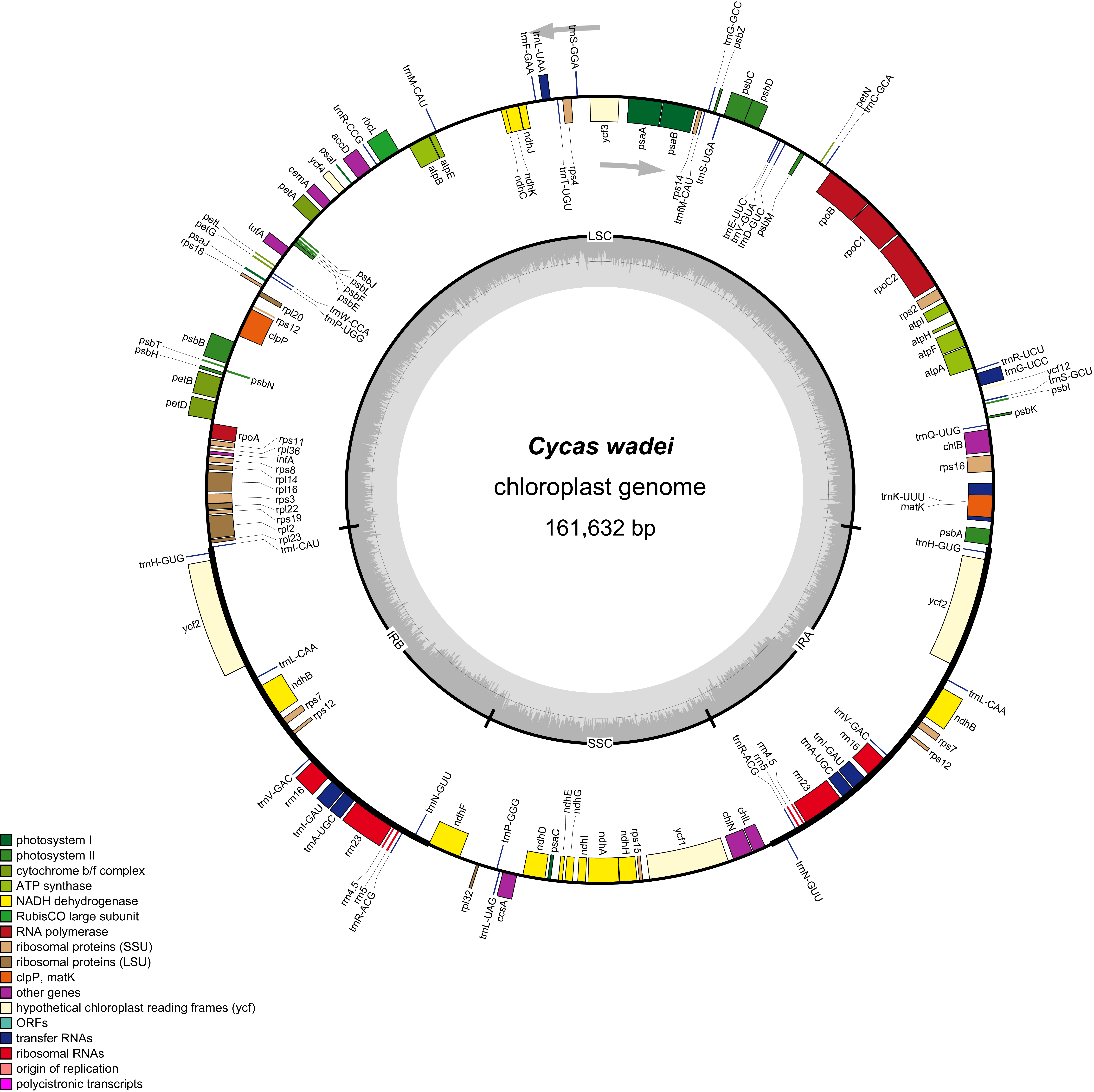


**Figure S2** Global alignment of 11 *Cycas* genomes and using mVISTA. Alignment was performed using *C. aenigma* as a reference. Grey arrows above the alignment indicate the orientation of genes. Purple bars represent exons, blue ones represent introns, and pink ones represent non-coding sequences (CNS). A cut-off of 50% identity was used for the plots. The Y-scale axis represents the percent identity within 50–100%.





**Figure S3** Comparison of inverted-repeat (IR) and single-copy (SC) borders among 11 *Cycas* chloroplast genomes from six sections. Gene annotation or portions are represented by colored boxes. JSA: junction between SSC and IRa; JSB: junction between SSC and IRb; JLA: junction between LSC and Ira; JLB: junction between LSC and IRb.


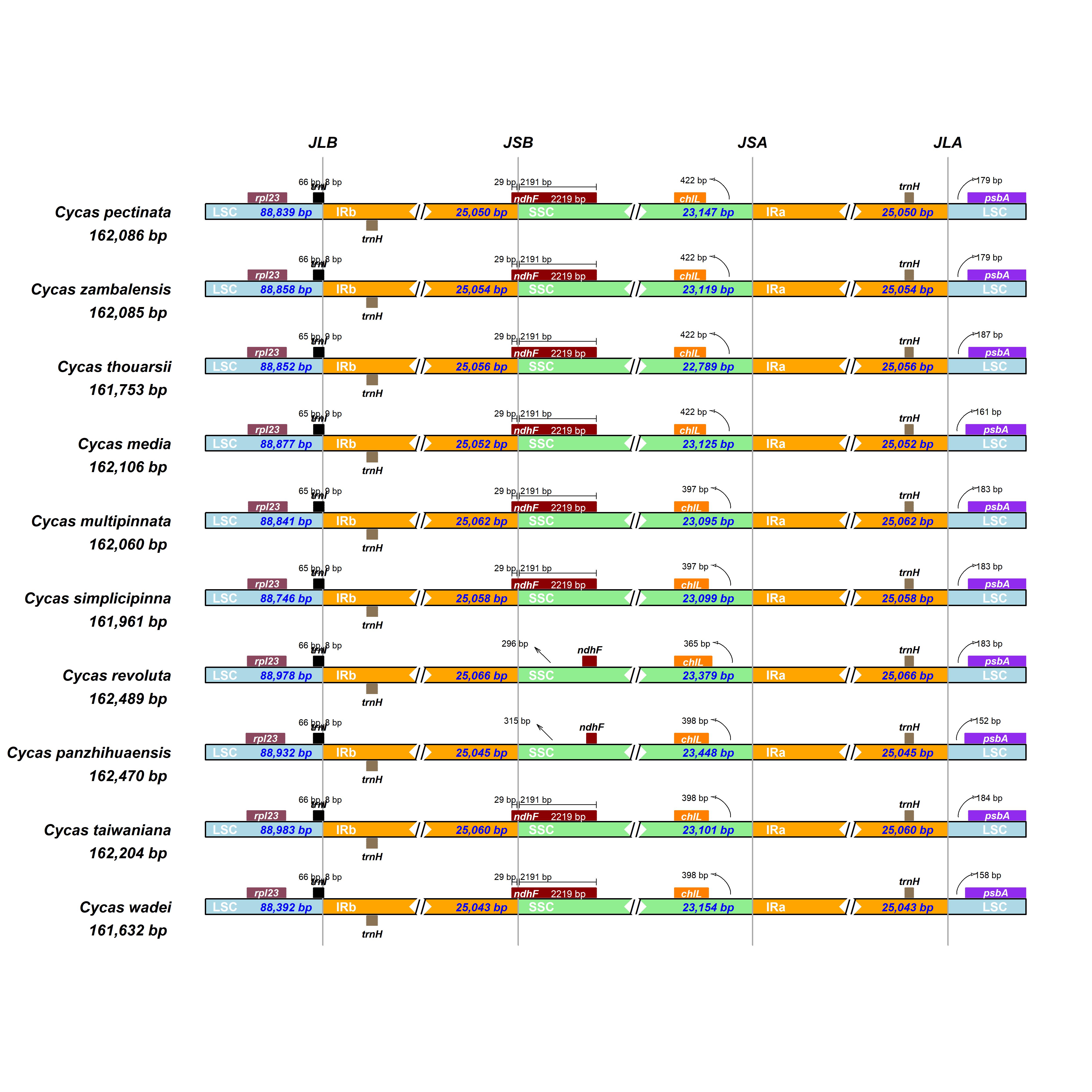


**Figure S4** The type and distribution of SSRs in the 47 *Cycas* chloroplast genomes. (a) The proportion of SSR distribution in different species (b) Number of identified SSR motifs in different repeat class types.


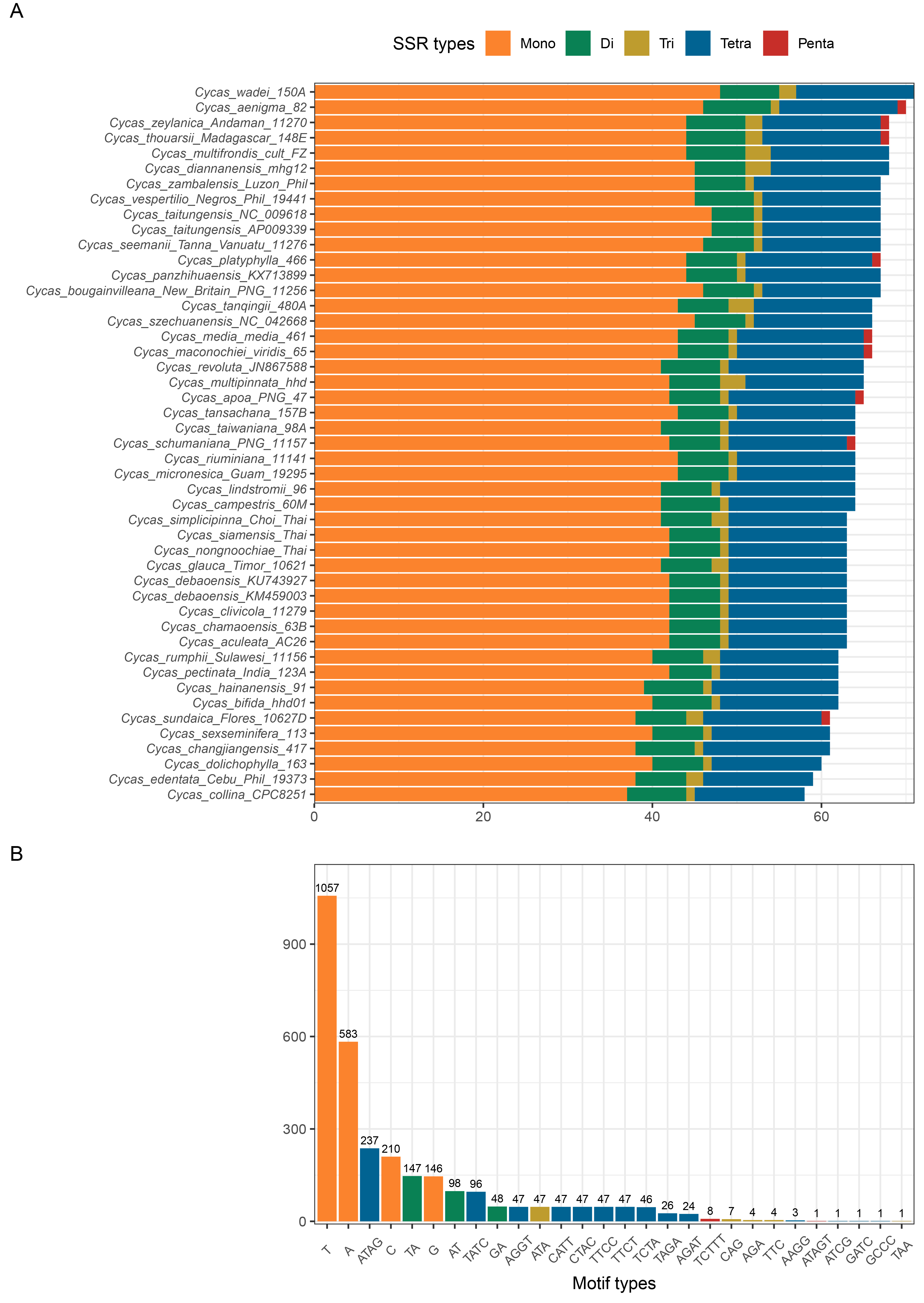


**Figure S5** Combined ML topology inferred by ASTRAL (species tree) for *Cycas*, with summary of conflicting and concordant genes. For each branch, the top number indicates the number of homologs concordant with the species tree at that node, and the bottom number indicates the number of homologs in conflict with that clade in the species tree. The pie charts at each node present the proportion of homologs that support that clade (blue), the proportion that support the main alternative for that clade (pink), the proportion that support the remaining alternatives (orange), and the proportion that inform (conflict or support) this clade that have no bootstrap support (grey).

**
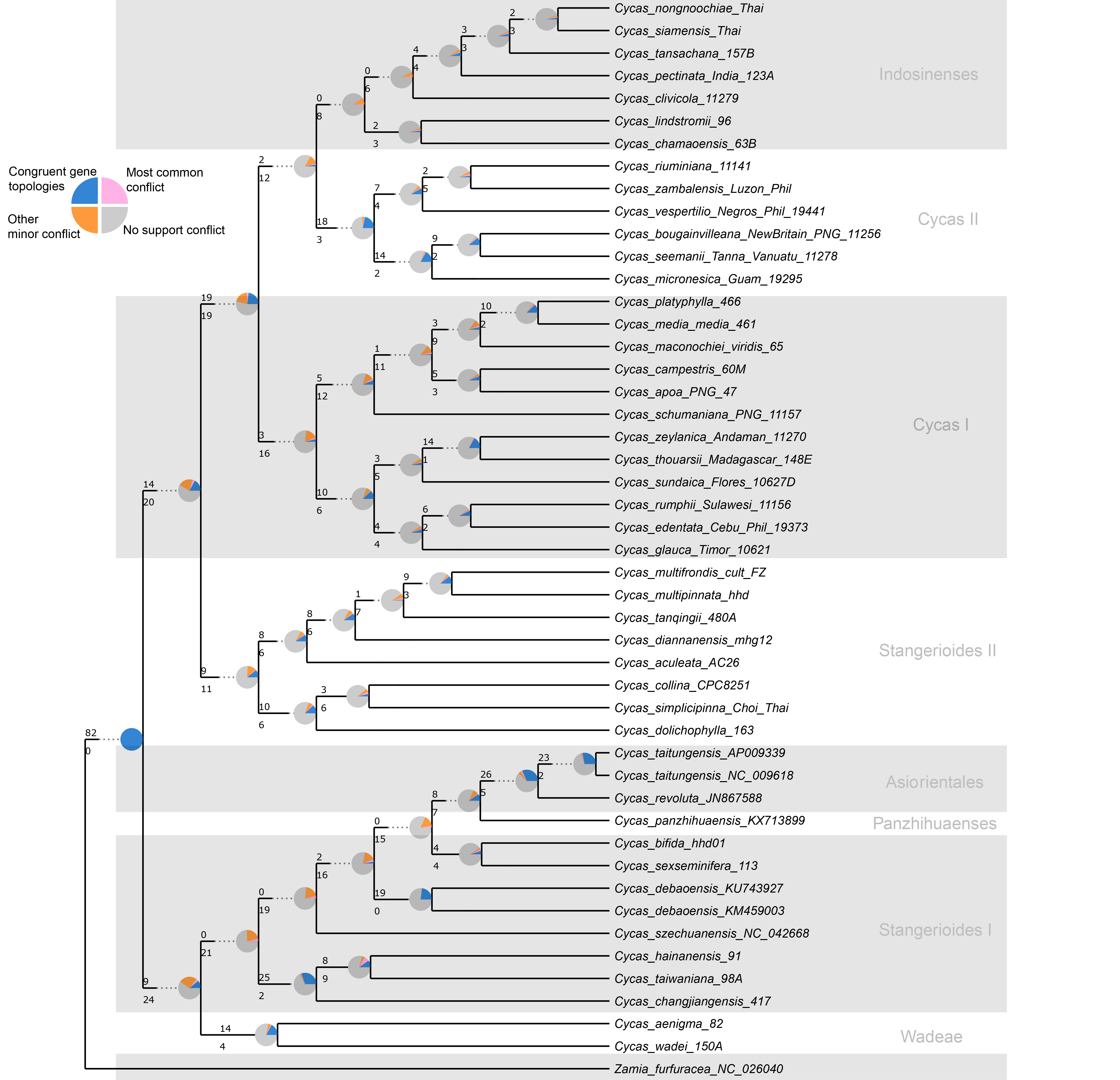
**

**Figure S6** Principal coordinate analyses depicting ordinations of rooted tree topologies (Robinson-Foulds) of four species trees versus six gene-cluster trees. (a): Plots for the first two principal coordinates; (b) Plots for the first and third principal coordinates. In both plots, a total of 10 trees were obtained from different phylogenetic inference methods (ML: maximum likelihood, BY: Bayesian method) based on all protein-coding genes (Concatenate_ML: inferred ML tree based on concatenated genes; Concatenate_BY: inferred Bayesian tree based on concatenated genes; Astral_ML: inferred species tree using ASTRAL-III based on ML gene trees, Astral_BY: inferred species tree using ASTRAL-III based on Bayesian gene trees), and different gene tree clusters (Clusters 1–3) are plotted. The inset dendrograms reflect the tree distances between the species tree and the cluster trees revealed by TREESPACE.


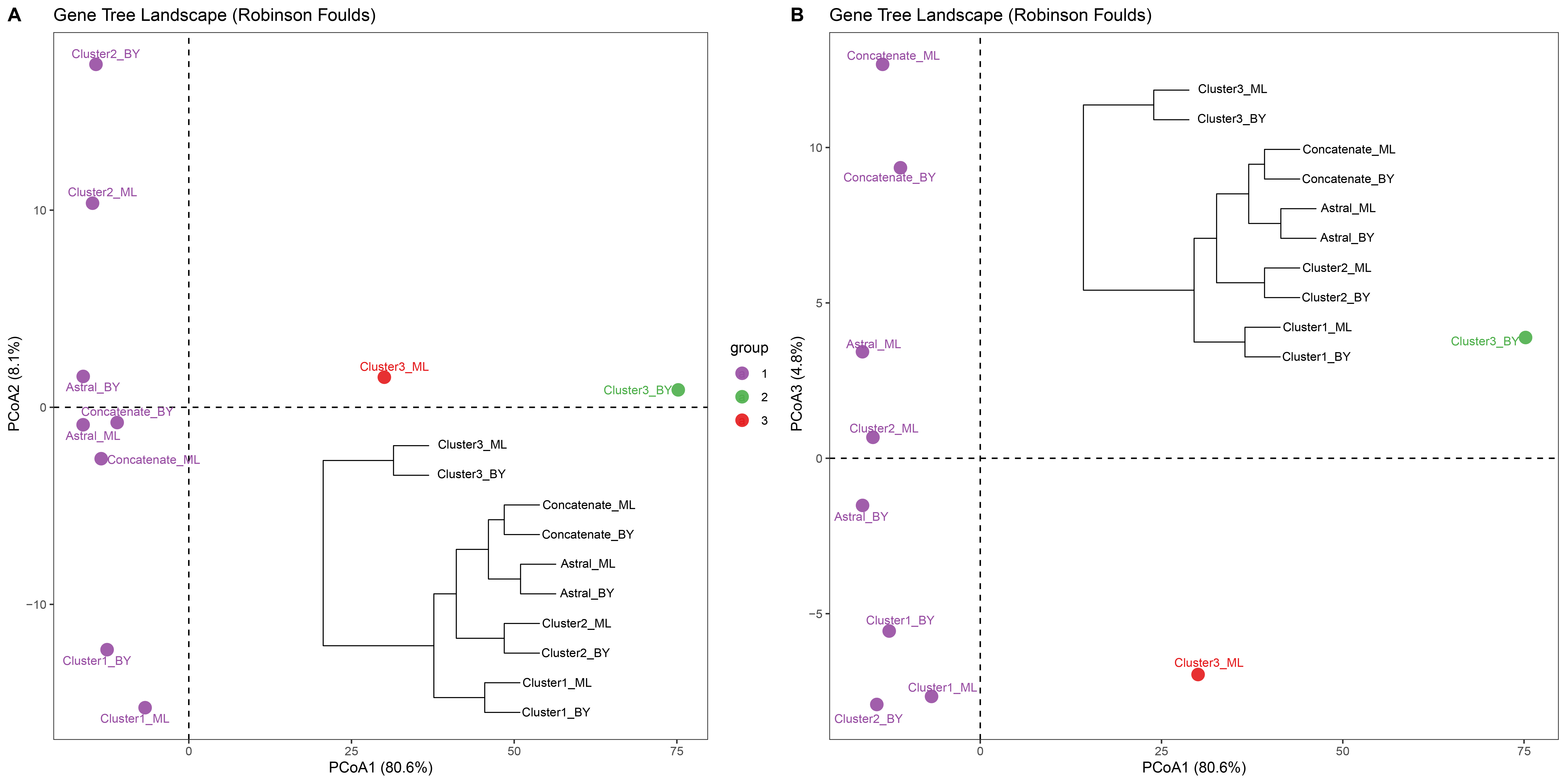


**Figure S7** Inferred phylogenies of *Cycas* using RaxML based on different concatenated plastid gene clusters obtained by Maximum likelihood and Bayesian gene trees (clusters 1-3, see Table 1 for the genes grouped by different methods).





**Figure S8** Principal coordinate analyses depicting ordinations of rooted tree topologies (Robinson-Foulds) of two species trees (ASTRAL species tree and concatenated gene tree) versus 11 gene trees based on different gene functional groups (see Figure 2 and Table S3 for the group information). (a): Plots for the first two principal coordinates; (b) Plots for the first and third principal coordinates.


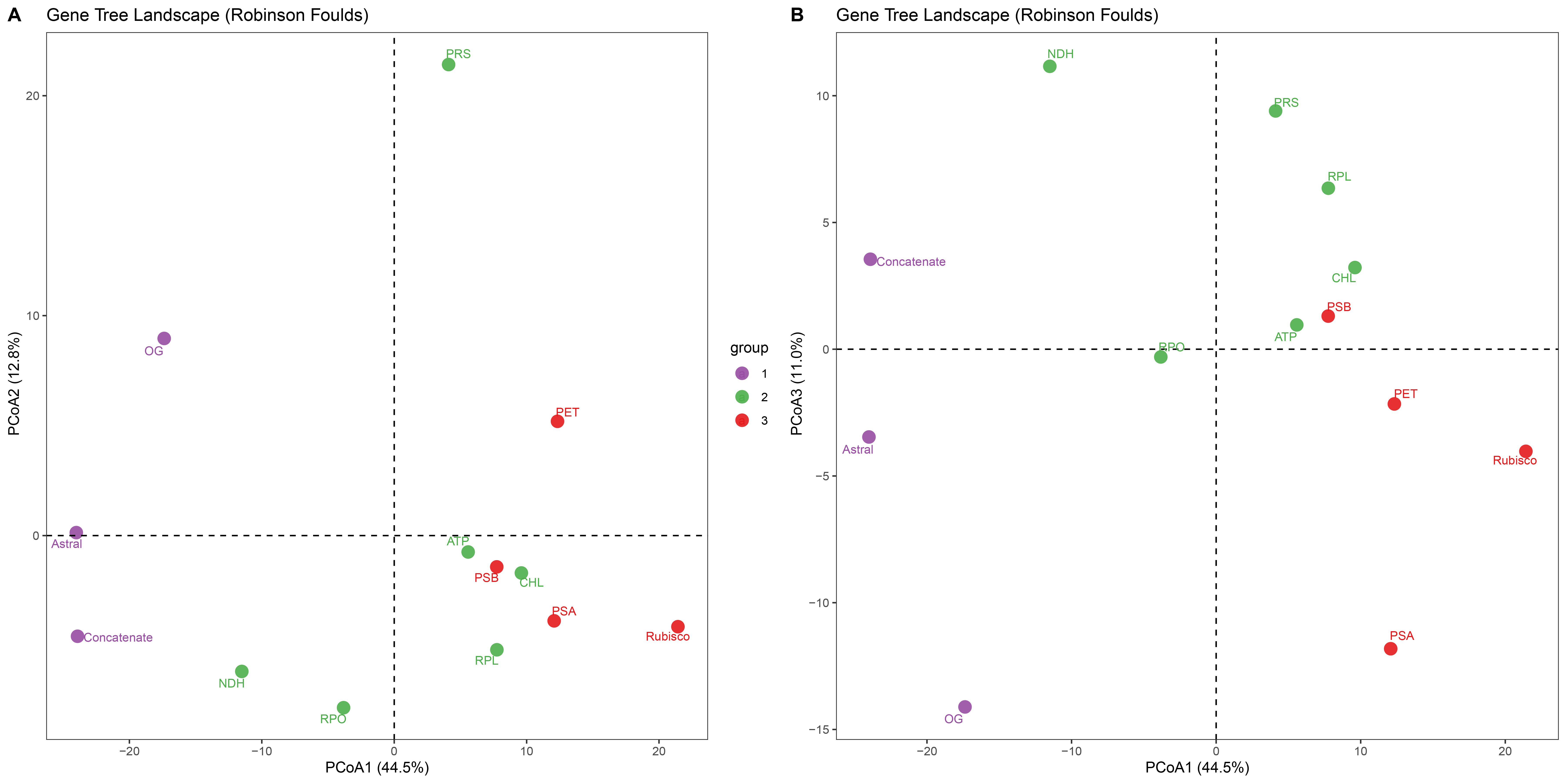


**Figure S9** Inferred phylogenies of *Cycas* using RaxML based on different concatenated functional gene groups (see Figure 2 and Table S3 for the information of 11 genes groups). The three indicated groups correspond to the clusters revealed in Fig. S8.
